# Neuron and astrocyte aggregation and sorting in three-dimensional neuronal constructs

**DOI:** 10.1038/s42003-021-02104-2

**Published:** 2021-05-17

**Authors:** Md Fayad Hasan, Yevgeny Berdichevsky

**Affiliations:** 1 Department of Electrical and Computer Engineering, Lehigh University, Bethlehem, PA USA; 2 Department of Bioengineering, Lehigh University, Bethlehem, PA USA

**Keywords:** Neurological models, Neuronal physiology, Astrocyte, Diseases of the nervous system, Neurological disorders

## Abstract

Aggregation and self-sorting of cells in three dimensional cultures have been described for non-neuronal cells. Despite increased interest in engineered neural tissues for treating brain injury or for modeling neurological disorders in vitro, little data is available on collective cell movements in neuronal aggregates. Migration and sorting of cells may alter these constructs’ morphology and, therefore, the function of their neural circuitry. In this work, linear, adhered rat and human 3D neuronal-astrocyte cultures were developed to enable the study of aggregation and sorting of these cells. An in silico model of the contraction, clustering, and cell sorting in the 3D cultures was also developed. Experiments and computational modeling showed that aggregation was mainly a neuron mediated process, and formation of astrocyte-rich sheaths in 3D cultures depended on differential attraction between neurons and astrocytes. In silico model predicted formation of self-assembled neuronal layers in disk-shaped 3D cultures. Neuronal activity patterns were found to correlate with local morphological differences. This model of neuronal and astrocyte aggregation and sorting may benefit future design of neuronal constructs.

## Abstract (web summary)

Hasan and Berdichevsky report the use of 3D neuronal-astrocyte co-cultures in PDMS confinement to enable the study of aggregation, migration, contractility, and sorting of these cells. They show that aggregation is a neuron mediated process, and formation of astrocyte-rich sheaths in 3D cultures depends on differential attraction between neurons and astrocytes, highlighting the use of this model in future design of neuronal constructs.

## Introduction

In the developing mammalian brain, newly specified neurons migrate over long distances before they differentiate and form synapses^1^. This migration is tightly orchestrated by the presence of guidance clues in the extracellular environment, and results in the complex, layered microarchitecture of the adult brain. Injury to the brain, including trauma, stroke, or resection surgery to remove a tumor or epilepsy focus, may create a fluid-filled cavity^2–4^. Imaging data from patients show that cavity size may change dynamically, suggesting that active cell proliferation, apoptosis/necrosis, and collective cell movement may be occurring in the absence of normal developmental clues^5,6^. Collective movements of cells, termed collective cell migration, have been described for non-neuronal cells during organ development, tumor invasion, and wound healing^7^. It has also been described for astrocytes in the context of brain injury, which is frequently characterized by glial scarring: presence of high numbers of reactive astrocytes and microglia close to the injury site^8–10^. Cells also undergo spontaneous aggregation. Dissociated cells in vitro aggregate into spheroids^11^. Neurospheroids aggregated from neuronal stem cells or progenitors are used in a variety of in vitro assays^12,13^. A mixture of neuronal cells injected into a lesion cavity of a rat model of stroke assembled into rosettes with layer-like structure^14^. In our previous work, neurons in three-dimensional (3D) aggregates in vitro self-assembled into ring-like layers^15^.

Self-organization of cells into layered 3D aggregates, termed cell sorting or tissue segregation, has been described for non-neuronal cell types^16^. It may arise from differential cell adhesion, although cell contractility and migration may also play a role^17^. However, to the best of our knowledge, there are no reports of aggregation and collective migration of neurons and astrocytes in a 3D environment. This behavior of neuronal cells may be an important component of brain’s response to injury. It may also be an important consideration in the engineering of 3D neuronal constructs. Implantation of engineered tissues into brain cavities resulting from stroke or from traumatic brain injury has been proposed as a means to improve patients’ cognition and life quality^18^. In vitro brain tissue constructs, especially when populated by human cells, represent a promising tool for drug development that may not suffer from replication issues of animal models^19^. Collective migration and sorting of cells in these constructs may alter their morphology and, therefore, their function, as neural circuit operation is heavily dependent on the arrangement of neurons^20,21^. It may be possible to manipulate cell interactions to guide aggregation and collective migration to create constructs with desired structure^11,22^, and obtain better results in patients or in vitro models.

Previous approaches to creation of neural aggregates in vitro utilized immature cells such as neuronal stem cells or induced pluripotent stem cells (iPSCs). The aggregates were then differentiated leading to creation of brain or cortical organoids^23,24^. Organoids suffer from poorly controlled size due to cell proliferation, and from poor replicability and cell type specification^25^. We have previously determined that neural aggregates can be constructed from differentiated, postmitotic neurons and differentiated astrocytes^15^. Disc-like aggregates created in our previous work were characterized by high replicability of size and cell type composition. However, the following questions remained open:

1. How does 3D construct shape influence neuronal layer self-assembly and neural processes?
2. What relative roles do neurons and astrocytes play in governing the morphology and shape of 3D constructs?
3. Is there a relationship between construct morphology and neuronal activity?

**Fig. 1:**
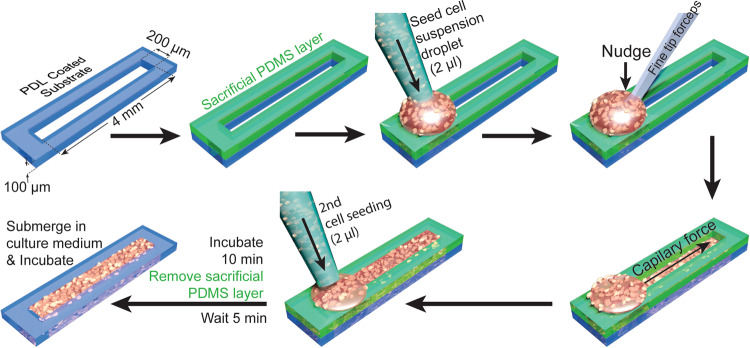
Workflow for creating linear 3D cultures. PDMS device with slit is cut out from a 100 μm high PDMS film and plated on cell culture substrate. An identical device is stacked on top of the base device. Dense cell suspension is then seeded at the edge of the slit. Cell suspension will not go in slit due to surface tension. A nudge with a fine forceps tip breaks surface tension and then slit’s capillary force pulls the cell suspension in. In total, 2 μl cell suspension is again seeded at the previous spot to ensure complete filling of the slit. The device is then incubated for 10 min and sacrificial PDMS layer is removed. After another 5 min of incubation, the whole device is submerged with culture medium and maintained for up to 18 days as described in “Methods”.

In this work, we developed elongated 3D aggregates (Fig. 1) that enabled us to address these questions, and to develop a predictive model of the collective cell behavior.

## Results

### Spontaneous cluster formation in linear 3D cultures

**Fig. 2:**
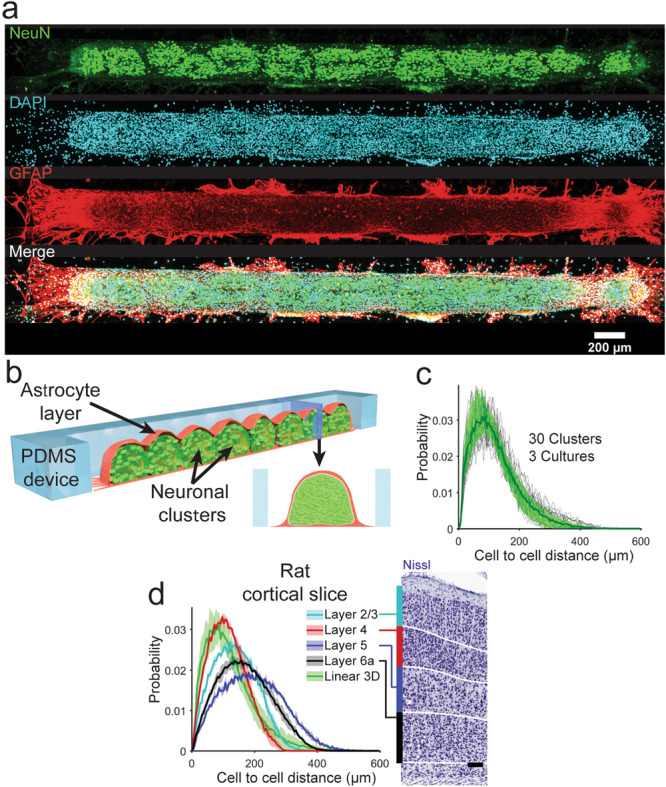
Immunohistochemistry analysis of linear 3D culture and comparison with rat cortical section. **a** Projection of brightest point of image stack of linear 3D culture, fixed on DIV 14, co-stained with (from top to bottom) NeuN (green), DAPI (cyan), and GFAP (red). Bottom row shows merged image. **b** Representation (not drawn to scale) of linear 3D culture shows neuronal clusters and superficial astrocyte layer. (Red: astrocytic layer, Green: Neuronal layer) **c** Cell to cell distance profile (CCDP) counted from NeuN^+^ cells of 30 clusters from three cultures (11, 8, and 11 clusters). Thin black lines show CCDP of individual clusters, thick green line indicates the mean CCDP of all clusters with standard deviation indicated by green shaded area. **d** CCDPs calculated from different layers of Nissl stained rat cortical slices from PND 08 rat on the left, Nissl stained image and layer labels on the right, image courtesy: BrainMaps: An Interactive Multiresolution Brain Atlas; http://brainmaps.org. Thick line for each layer (color indicated in legend) shows mean of CCDPs from six regions of interests (ROI) from three *z*-positions (one from each hemisphere of each *z*-position) while shaded area of same color indicates the standard deviation. CCPD for linear 3D cultures is included for comparison. Scale bar: 100 μm for **d** (right).

Dense dissociated neonatal rat cortical cell solution (40 million cells/ml) was seeded in a 200 μm × 4 mm slit in a 100 μm high polydimethylsiloxane (PDMS) device (Fig. 1, see “Method” for details). Cells self-assembled into a 3D aggregate (Supplementary Video 1 and Fig. 2a, b) over 8 days of incubation. Upon staining these cultures for neuronal soma (NeuN), astrocyte (GFAP), and nuclear (DAPI) markers, we observed clearly distinguishable neuronal (NeuN^+^) clusters and bright GFAP^+^ astrocytic superficial layer (Fig. 2a, b). We then determined the Cell to Cell Distance Profile (CCDP) for each cluster in 3D aggregates. CCDP showed the probability of two randomly selected neurons to be at a specific distance apart. CCDPs of 30 clusters form three cultures showed similar CCDP (Fig. 2c, thick green line represents the mean, while shaded region represents the standard deviation (std)). To compare neuron spacing in our linear 3D cultures to the intact cortex, we analyzed Nissl stained rat cortical sections obtained from BrainMaps: An Interactive Multiresolution Brain Atlas^26^. Densest cortical layer 4 had a CCDP that was similar to linear 3D culture CCDP (Fig. 2d, root mean square errors between CCDP of Linear 3D culture (Green) and CCDP of Layer 2/3 (Cyan), layer 4 (Red), layer 5 (Blue), and layer 6 (Black) were 0.0017, 0.00082, 0.0018, and 0.0025, respectively). The peaks of the mean of CCDP, indicative of the most probable distance between two neurons, in cortical layer 4 and in vitro linear 3D culture were at 92.5 and 72.5 μm, respectively. Both were substantially larger than the diameter of a neuronal soma (14 μm in rat brain^27^, and 15.3 ± 2.3 μm in our linear 3D cultures, *n* = 300 neurons). This indicated that both in the densest layer of the intact cortex and in our linear 3D cultures, neuronal somas were not in close contact. Space between neuronal soma is filled mostly with dendrites, axons, synapses, and astrocytic processes in the intact cortex^28^, and the same may be true of our 3D cultures.

### Spontaneous alignment of neuronal and astrocytic processes

**Fig. 3:**
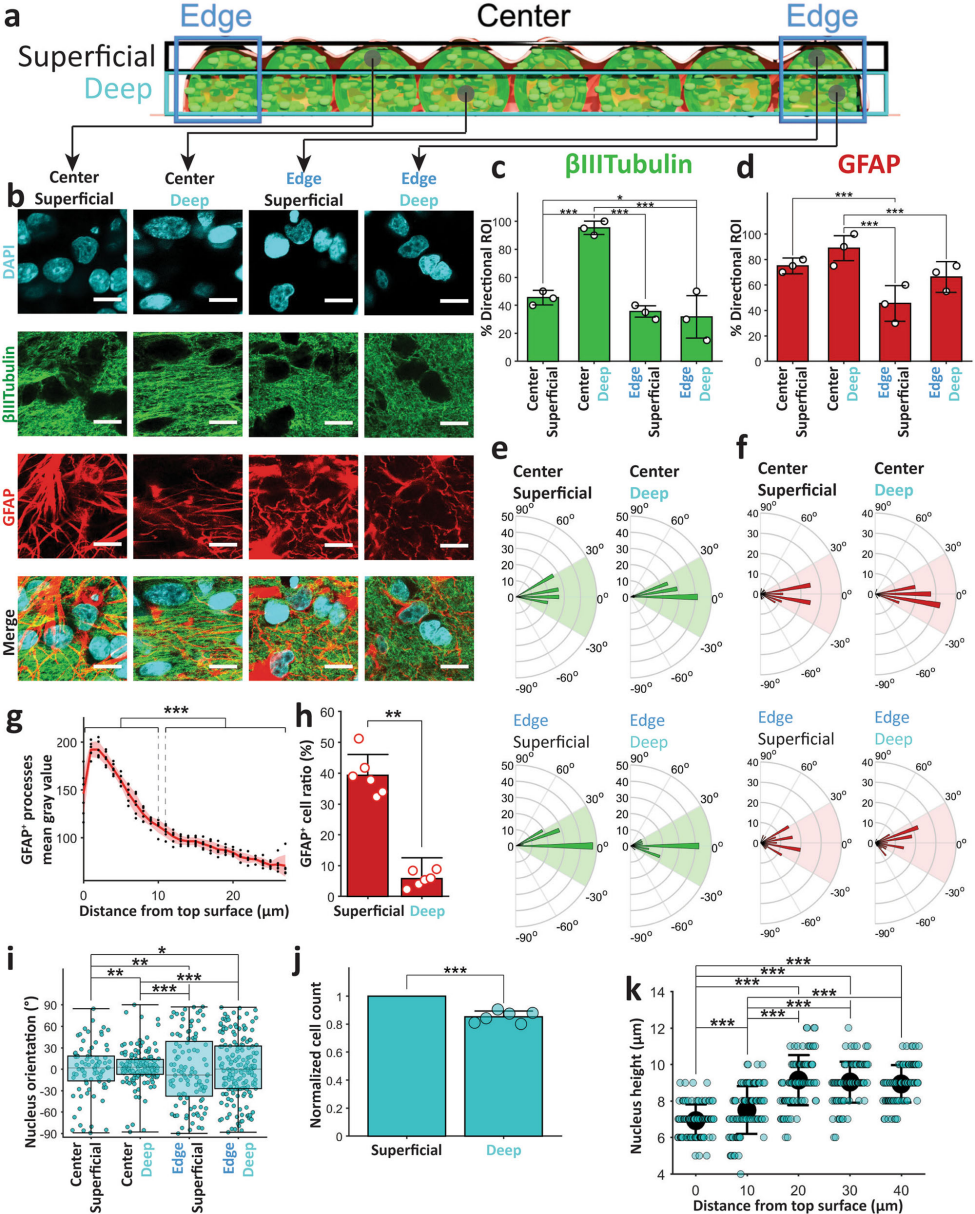
Processes and nucleus alignment. **a** Schematic of a linear 3D culture with edge (blue), superficial layer (black), and deep layer(cyan) marked with rectangles. Sample locations of regions of interest are marked with gray circles. **b** Regions of interest (ROIs) from confocal *z*-stack images from culture locations indicated in **a** (from top bottom: DAPI, βIII-Tubulin, GFAP and merge, scale bar 10 μm). Mean and standard deviation of % of ROIs with directionality (alignment) for βIII-Tubulin (**c**) and GFAP (**d**) stained images at locations specified on the *x-*axis. Polar plot histograms of the directions (−90° to 90° with 5° bin width) found in the ROIs with directionality (**c**, **d**) for βIII-Tubulin^+^ neuronal (**e**) and GFAP^+^ astrocytic processes (**f**) at different locations. Radial lines indicates angles between aligned processes and culture’s long axis, and concentric circles show numbers of ROIs with alignment angle within a bin range. Green (**e**) and red (**f**) shaded region shows the ±30° angle range from culture long axis. **g** Average gray value (black dots) with mean (red thick line), and standard deviation (red shaded area) of GFAP^+^ processes at different distances (indicated on the *x*-axis) from top surface at the center of culture. **h** Means ± standard deviations of the number of GFAP^+^ cells in the superficial layer (top 10 μm) and deep layer (15–25 μm deep). **i** Box plot of nucleus (DAPI^+^ objects) alignment angles at different locations indicated on the *x*-axis. **j** Normalized number of DAPI^+^ objects in superficial layer (top 10 μm) and deep layer (15–25 μm deep) at center locations. **k** Means (black filled circle) with standard deviations of nucleus bounding cuboid height (along *z* direction) at different distances from top surface at center locations. Same 60 ROIs for each location from three cultures (20 each) were analyzed for **c** and **d**. Among these ROIs, 27, 57, 21, and 19 ROIs showed βIII-Tubulin alignment, and 45, 53, 27, and 40 ROIs showed GFAP alignment at center superficial, center deep, edge superficial, and edge deep locations, respectively. These directional ROIs were analyzed in **e** and **f**. For **g**, **h**, **j**, same *n* = 6 (100 μm × 100 μm) confocal *z*-stacks (40 μm deep from top surface) from three cultures’ center locations (two each) were analyzed. For **k**, *n* = 90 DAPI^+^ objects for each distance from surface (from three cultures) were analyzed. In total, 30 DAPI+ objects were counted from each culture’s *z*-plane for each distance from surface in **k**. Asterisks indicate results of (**j**) paired-sample *t*-test and (**c**, **d**, **i**, and **k**) two-sample Kolmogorov–Smirnov test (*p < 0.05, **p < 0.01, and ***p < 0.001).

Next, we analyzed the morphology of neuronal (βIII-Tubulin^+^) and astrocytic (GFAP^+^) processes to determine if their alignment was impacted by the linear geometry of the 3D cultures. We imaged three linear 3D cultures on DIV 14 at four locations: (1) edge superficial, (2) edge deep, (3) center superficial, and (4) center deep, as indicated in Fig. 3a. Alignment between βIII-Tubulin^+^ processes (neurites) could be observed at center deep locations, but not at other locations (Fig. 3b). We quantified the alignment with 2D cross-correlation based technique (see “Methods” and Supplementary Fig. 1). With this method, we determined whether processes in a 10 μm × 10 μm region of interest (ROI) were aligned. If that were the case, we also determined the angle of alignment relative to the culture’s long axis. We found that almost all ROIs (95.3 ± 4.8%, mean ± std) were aligned at cultures’ center deep locations, but the percentage of aligned ROIs at other locations was significantly lower (Fig. 3c). Astrocytic processes were similarly aligned at center deep locations (Fig. 3b) with significantly higher number of ROIs with directional processes at center deep locations (88.9 ± 9.8%) than edge locations (Fig. 3d). Unlike neurites, astrocytic processes were more aligned at center superficial locations (75 ± 6.2%) than edge superficial locations (45.5 ± 14%), and alignment was not significantly different between center superficial and deep layers (Fig. 3d).

### Processes in aligned regions were aligned along the culture long axis

We observed that the ROIs with directionality were mostly aligned along the culture’s long axis as can be seen in the angle histograms (radius indicates the number of ROIs) of Fig. 3e, f, for neurites and astrocytic processes. For 78.8% center superficial, 97.7% center deep, 57.6% edge superficial, and 61% edge deep ROIs with directionality, neurites were aligned at an angle within ±30° of the culture’s long axis (Fig. 3e). For astrocytic processes, these percentages were 77.1%, 95.6%, 69.1%, and 62.1%, respectively (Fig. 3f) Interestingly, the center deep processes, with more directional ROIs, were also more aligned along the culture long axis than processes at other locations. Alignment of processes indicates that the 3D culture shape influences morphology of both neurons and astrocytes.

### Astrocyte-rich superficial layer

Images from three cultures’ centers (two imaging locations each) showed significantly brighter GFAP expression in astrocytic processes in the superficial 10 μm layer (grayscale value of 156.8 ± 27.9, mean ± std) than GFAP expression in the processes 10–25 μm below surface (87.7 ± 13.4, Fig. 3g). This indicated reactive nature of these superficial astrocytes^29,30^. Astrocyte (GFAP^+^ cell) percentage of total cell (DAPI^+^ object) count was also significantly higher in the superficial layer (39.3 ± 6.8%) than deeper layer (5.8 ± 2.5%, Fig. 3h). DAPI^+^ object counts showed consistently higher cell numbers in the superficial layer (240 ± 30 cells per ROI) than the layer below (197 ± 27 cells per ROI, Fig. 3j). However, this difference was much lower than the difference in percentage of astrocytes: 6.8 times more astrocytes vs. ~1.2 times more total cells in the superficial layer. These data suggest spontaneous sorting of astrocytes to superficial culture layer.

### Shape of cell nuclei is affected by 3D culture geometry

We analyzed nucleus alignment and shape to determine if they were affected by culture geometry. The height of nuclei (DAPI^+^ objects) along the *z*-axis (normal to culture’s base substrate) and the elongation direction in *X*–*Y* plane (parallel plane to culture’s base substrate) were calculated to determine the degree of alignment relative to culture’s long axis (see “Methods”). Nuclear alignment was highest in center-deep locations, followed in descending order by center-superficial, edge-deep, and edge-superficial locations (Fig. 3i). We also found a significant decrease in nucleus height in superficial layer compared to deep layer (Fig. 3k). This suggests that culture shape-induced alignment affects not only processes, but also cell nuclei, and that this mechanism is most active in center-deep culture locations.

### Astrocytes enhance neuronal activity

Next, to assess the role of astrocytes on spontaneous neuronal activity, we created three types of cultures: (1) control cultures from rat cortical cells, (2) cultures from rat cortical cells maintained in the presence of 3 μM Cytosine Arabinofuranoside (AraC), and (3) cultures from 85% dissociated rat cortical cells +15% rat astrocytes (obtained and propagated in vitro from an earlier dissection).

**Fig. 4:**
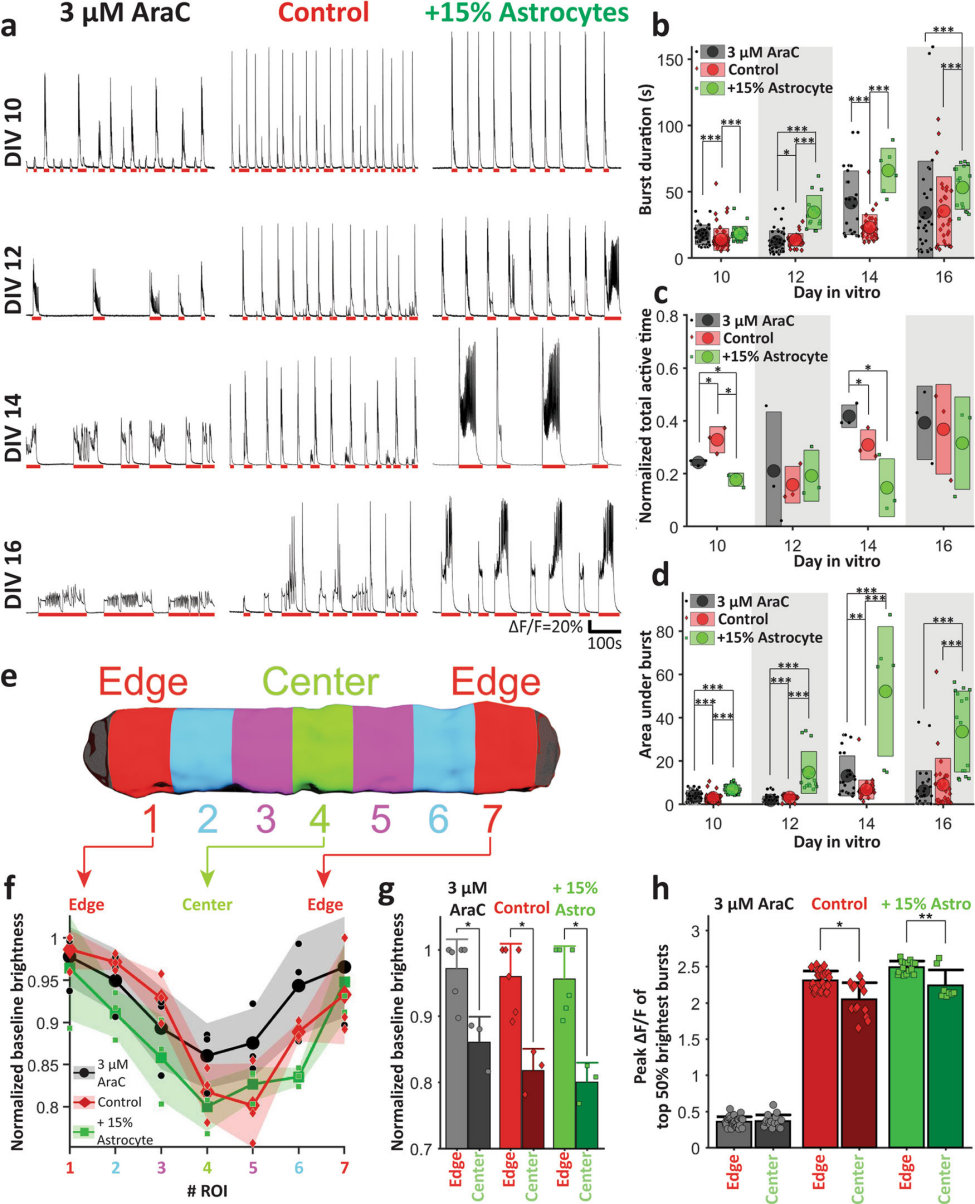
Activity analysis in linear 3D cultures. **a** Ca^2+^ activity in culture with chronically applied AraC (left), control culture (middle), and culture with +15% rat astrocytes (right) on (from top to bottom) DIV 10, 12, 14, and 16. Mean (circle) with standard deviation (rectangle) of **b** individual burst durations, **c** normalized total active time, and **d** area under curve of individual bursts on different DIVs. **e** Schematic of a culture with seven segments that were analyzed separately. **f** Normalized baseline brightness for each segments of different types of cultures on DIV 16. (Individual points indicate normalized baseline value for a specific type of culture on a specific DIV, large dotted solid lines indicate mean, shaded regions indicate standard deviation). **g** Mean with standard deviation of normalized baseline brightness of edge (segments 1 and 7) and center (segment 5) for different types of cultures on DIV 16. **h** Mean with standard deviation of peak brightness change (Δ*F*/*F*) during bursts at edge (segments 1 and 7) and center (segment 5) for different types of cultures on DIV 16. For **b** and **d**, for 3 μM AraC, Control and +15% astrocytes cultures, *n* = 37, 63, 26 on DIV 10, *n* = 46, 31, 15 on DIV 12, *n* = 27, 36, 6 on DIV 14, and *n* = 31, 28,16 on DIV 16, respectively. *n* is the number of bursts counted; three cultures were analyzed for each case. For **c**, *n* = 3 cultures for each case, for **g**, *n* = 6 for edges and *n* = 3 for centers, *n* being number of segments analyzed. Three cultures were analyzed (two edge and one center segment from each culture). For **h**, for 3 μM AraC *n* = 64, 31, for control *n* = 52, 28 and for +15% astrocytes *n* = 30, 16 for edge and center, respectively, *n* is the number of bursts. Six edge segments and three center segments were analyzed from three cultures (two edge segment and one center segment each). For **b**–**d**, **g**, **h** asterisks indicate results of two-sample Kolmogorov–Smirnov test (*p < 0.05, **p < 0.01, and ***p < 0.001).

The goal of this experiment was to decrease astrocyte proportion with mitotic inhibition with 3 μM AraC or to increase astrocyte proportion by adding more astrocytes, and then determine the effect on neuronal activity. We used a Ca^2+^ indicator jRGECO1a^31^ expressed in neurons to detect activity. Neuronal activity causes dynamic changes in intracellular [Ca^2+^], leading to corresponding changes in the fluorescence of jRGECO1a expressing neurons^31^. We imaged three of each culture type on DIV 10, 12, 14, and 16. All types of cultures showed synchronized bursts across the whole culture starting from DIV 10 (red lines indicate detected bursts in Fig. 4a). Burst duration increased for all types of culture with DIV (Fig. 4a, b). Cultures with +15% astrocytes had significantly longer burst durations (34.6 ± 12.7, 66 ± 16.7, and 53.3 ± 16.5 s, mean ± std) than control (13.8 ± 4.7, 23.2 ± 9.7, and 35.5 ± 25.9 s) on DIV 12, 14, and 16, respectively (Fig. 4b). However, the total active time was lower for +15% astrocyte cultures compared to controls on DIV 10 and DIV 14 (Fig. 4c). To further compare activity, we measured the area under the curve (integration of Δ*F*/*F* with time) for each burst. The areas under burst in Fig. 4a for +15% astrocytes cultures (7 ± 2.1, 14.8 ± 9.7, 52.2 ± 29.9, and 33.6 ± 18.7) was significantly higher than for control (3 ± 1.7, 3.2 ± 1.2, 6.9 ± 4.4, and 9.1 ± 12.2) and 3 μM AraC cultures (3.9 ± 2.5, 1.9 ± 1.9, 13.1 ± 9.3, and 6.2 ± 9.3) for DIV 10, 12, 14, and 16, respectively (Fig. 4d). Addition of astrocytes thus significantly increased burst duration and intensity, as expected^32^. The effect of AraC was less clear. It decreased peak Δ*F*/*F* (Fig. 4h), but increased burst duration on DIV 10, 12, and 14 (Fig. 4b) and area under burst on DIV 14 (Fig. 4d), while decreasing total active time on DIV 10, but increasing it on DIV 14 relative to controls (Fig. 4c). This may be due to direct effects of AraC on neurons in addition to its antiproliferative effect on astrocytes.

### Location-specific differences in [Ca^2+^]

We then examined location-specific differences in neuronal activity on DIV 16 for all three types of cultures (three cultures each). The cultures were divided into seven segments as shown in Fig. 4e. During inactive periods, the mean normalized baseline fluorescence, proportional to neuronal baseline [Ca^2+^], of these segments (normalized with respect to the mean highest baseline among all of that culture’s sections) was calculated. For all three types of cultures (control, 3 μM AraC and +15% astrocytes), the baseline fluorescence gradually decreased (Fig. 4f) from edge to center. The edge segments (normalized baseline brightness: 0.97 ± 0.04, 0.95 ± 0.05, and 0.96 ± 0.05) exhibited significantly higher baseline [Ca^2+^] than center segments (0.86 ± 0.04, 0.8 ± 0.03, and 0.82 ± 0.3) in 3 μM AraC, control, and +15% astrocytes cultures, respectively (Fig. 4f, g). This indicated that resting [Ca^2+^] was higher at the edges compared to the center of all three types of linear 3D cultures.

We then investigated the peak brightness change (maximum Δ*F*/*F*) during bursts from different culture segments (Fig. 4h). In control and +15% astrocyte cultures, the edge segments exhibited significantly higher maximum Δ*F*/*F* (2.3 ± 0.1 and 2.5 ± 0.1, respectively) than center segments (2.0 ± 0.2 and 2.2 ± 0.2, respectively) during bursts. AraC significantly decreased peak Δ*F*/*F* both at center and edge (0.4 ± 0.1 and 0.4 ± 0.1, respectively). These results show that differences in alignment of neuronal and astrocytic processes between center and edge locations correlate with differences in baseline and dynamic [Ca^2+^] levels. Antiproliferative effect on astrocytes may be the reason for lack of differences between edge and center locations in AraC-treated cultures, although direct effect of AraC on neurons may also play a role.

### Bursts predominantly initiate in the center of linear 3D cultures

**Fig. 5:**
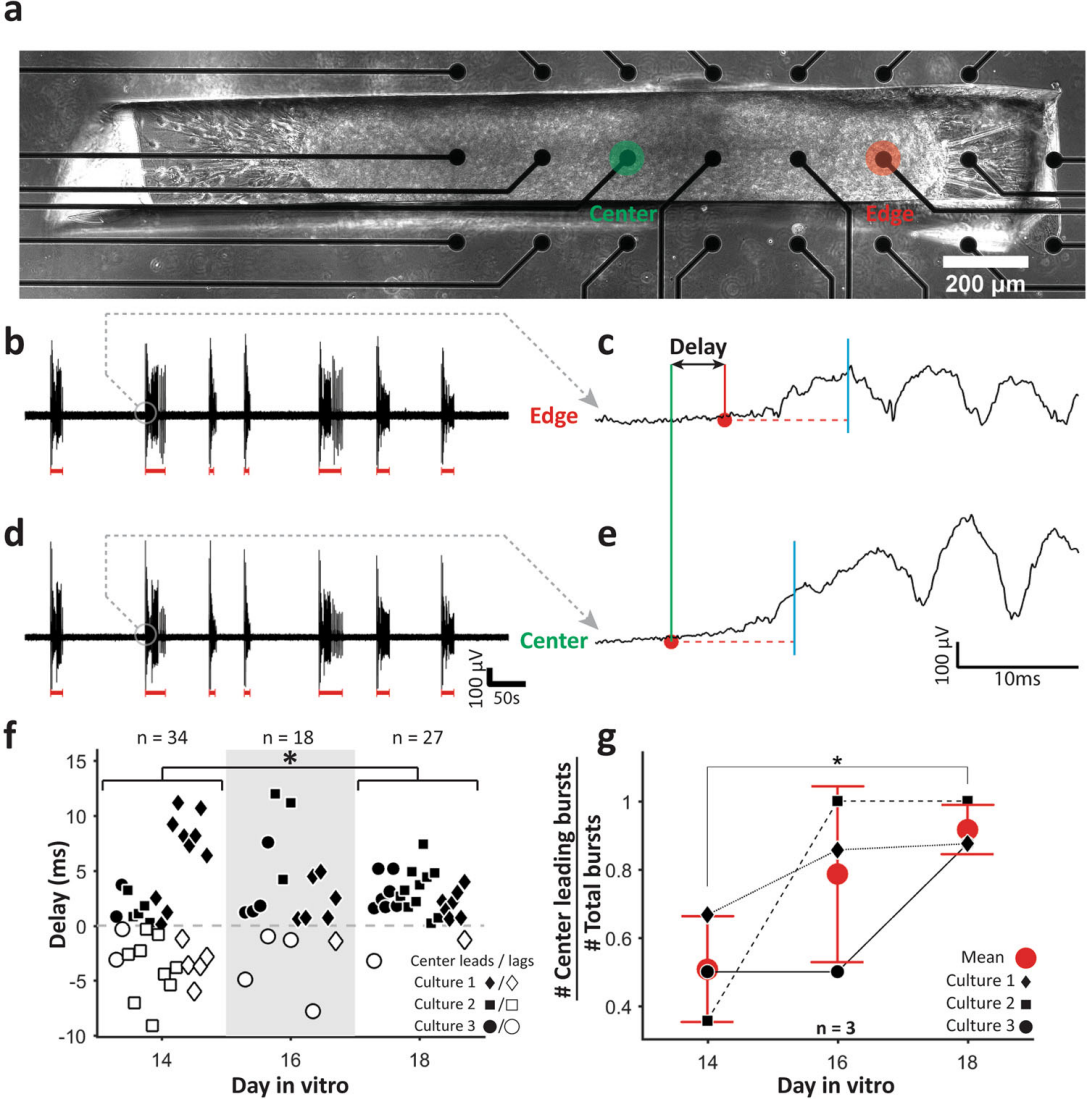
Local field potential (LFP) analysis. **a** Phase contrast image of a 3D linear culture on a multi-electrode array. Active channels at edge (red) and center (green) are marked by text and filled circles. Recorded LFPs from edge (**b**) and, center (**d**) electrode with detected bursts indicated by red lines. Zoomed in traces for the same burst (marked by gray circles in **b** and **d** from edge (**c**) and center (**e**) electrodes, respectively. Red circles indicate bursts’ initiation times. Red horizontal dashed line indicates threshold, cyan vertical line after threshold line indicates the time point (10 ms after trace crosses threshold) until which the trace must remain above threshold (red dashed line) for the initiation time to be registered. **f** Beeswarm plots of delays between burst initiations at edge and at center on different DIVs. Black filled and white filled shapes indicate that burst first initiated at the center or at the edge, respectively. **g** Mean ± standard deviation of the ratio of bursts initiating at the center (center leading bursts) and total detected bursts on different DIVs. Three cultures were recorded for all panels. For **f** and **g**, asterisks indicate results of two-sample Kolmogorov–Smirnov test (*p < 0.05, **p < 0.01, and ***p < 0.001).

To analyze burst initiation, we cultured linear 3D cultures on a 64 channel microelectrode array (MEA). Local field potential recordings from edge electrode (Fig. 5a, red filled circle) and center electrode (Fig. 5a, green filled circle) were analyzed. Recording showed bursts (red lines in Fig. 5b, d) similar to those observed with [Ca^2+^] imaging (Fig. 4a). We determined burst initiation times at edge and at center electrodes for each burst and determined the delay in initiation between center and edge as shown in Fig. 5c, e (also see “Methods”). On DIV 14, the bursts initiated both at center and edge (50 ± 15% bursts initiated at center) with no significant preference (Fig. 5f, g). However, as cultures matured, burst initiation point shifted toward the center of the linear 3D cultures. On DIV 18, almost all bursts (92 ± 7%) initiated at the center (Fig. 5f, g).

### In silico model of neuron and astrocyte aggregation

**Fig. 6:**
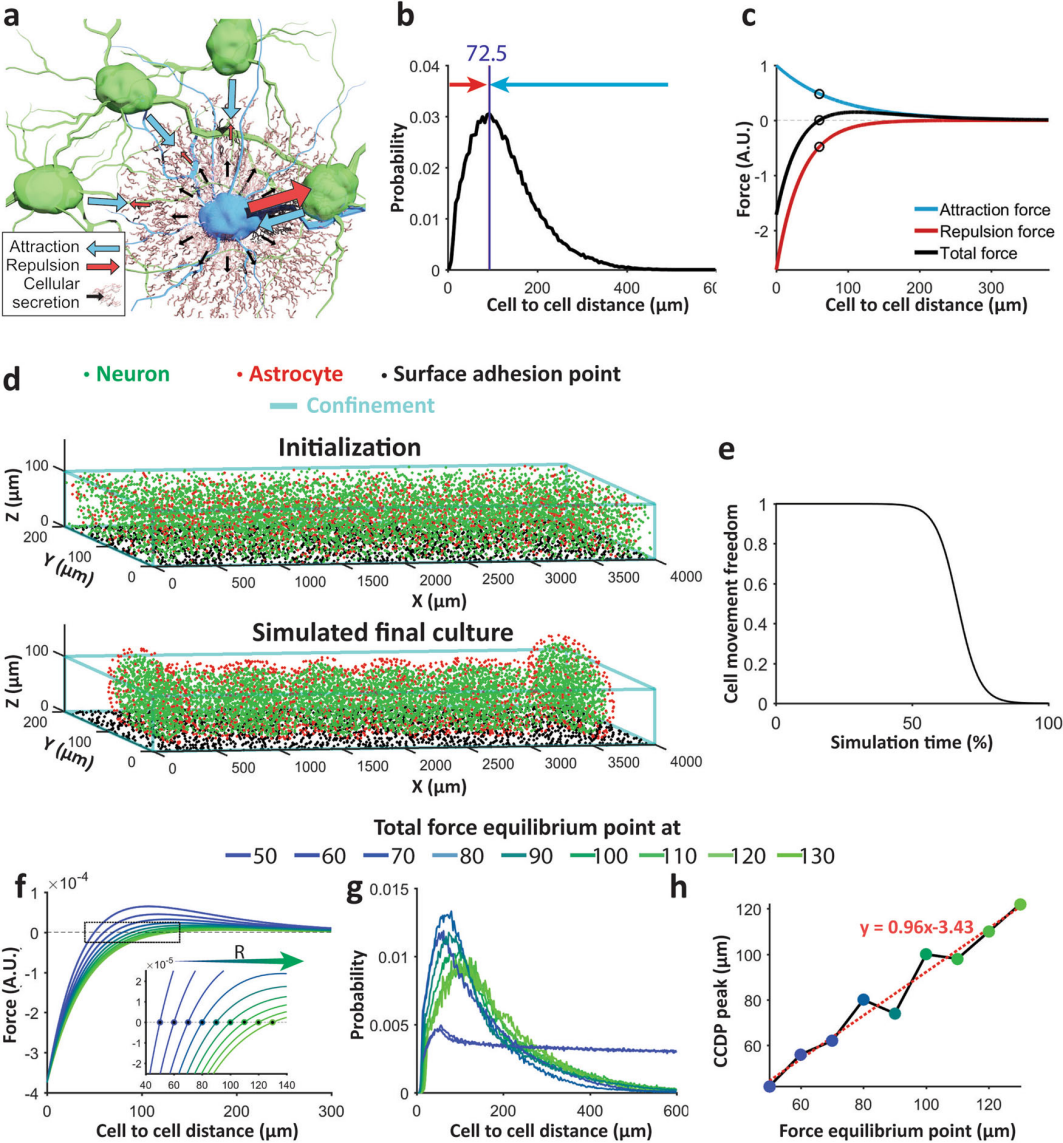
In silico model. **a** Schematic representation of attraction (blue arrow) and repulsion (red arrow) forces exerted by blue cell on green cells. Black arrow and brown lattice show molecules (ECM, growth factors, etc.) secreted by blue cell. **b** Mean cell to cell distance profile (CCDP) of 30 clusters from three linear 3D cultures. **c** Model attraction (blue) and repulsion (red) forces and their resultant total force (black). Black circles indicate force equilibrium point where attraction and repulsion forces are equal. **d** Random cells in confinement before simulation (top), simulated culture (bottom) clearly shows astrocytic superficial layer and clusters after reaching steady state. **e** Culture stiffness curve. **f** Total force curve with varying equilibrium point (zero-crossing) for different simulations. (Inset shows zoomed in view of the area indicated by black rectangle). **g** Mean CCDP of the clusters in simulated final culture with different total force equilibrium points. No clusters were formed for total force equilibrium point at 50 and 60 μm, and the cell to cell distance of entire culture was used to calculate the CCDP (traces with small peak and plateaued long tail). 8, 7, 6, 6, 6, 7, and 8 clusters were formed and CCDP of these clusters were used to calculate mean CCDP for total force equilibrium at 70, 80, 90, 100, 110, 120, and 130 μm respectively. **h** Distance of total force curve equilibrium point (zero-crossing from **f**) vs peak of mean CCDP (maximum point of each curve of **g**) shows linear relationship. Dashed red line indicates fitted line through the points and red text indicates fitted line equation.

We observed that neurons and astrocytes placed into a PDMS slit gradually aggregate into a 3D culture over first 8 days of incubation (Supplementary Video 1). These 3D cultures then contract until reaching steady-state length on ~DIV 12. We also observed that, in both steady-state 3D cultures and in the intact cortex, neurons maintain a minimum distance from each other. This distance is significantly larger than the diameter of a neuronal soma (Fig. 2c). We therefore hypothesized that below the distance at which CCDP peak occurs, neurons repulse each other and above this distance, neurons attract each other (Fig. 6a, b). Since these forces are exerted at relatively long-range, direct soma-to-soma contacts are unlikely to be responsible. These forces are likely to be generated by axons and dendrites sprouted by neurons. These processes express cell adhesion molecules, thus exerting an attractive force at relatively long ranges. They also occupy a proportionally higher volume of extracellular space with decreasing distance to the originating soma, thus exerting a repulsive force. Interaction between neurons may thus be attractive at long ranges, but repulsive at close ranges where cells cannot move closer to each other due to lack of space between processes. Cortical cells secrete extracellular matrix molecules, which may play a role in cell attraction or repulsion, as well as growth factors and other molecules which may influence cell migration and can also contribute to attractive or repulsive forces. We hypothesized that net attraction or repulsion is the result of two opposing forces that cancel each other at CCDP peak (Fig. 6c), resulting in a high probability of cell-to-cell distances having this value (72.5 μm in our cultures). We modeled these two forces with simple exponential functions of opposite signs (positive: attraction, and negative: repulsion, as shown in Fig. 6c). Additional forces acting on cells include gravity (constant downward force) and the surface adhesion of PDL-coated culture substrate. We implemented an aggregation model based on these forces in silico (see “Methods”).

We simulated randomly placed 6400 neurons and 1600 astrocytes (cell numbers were selected to roughly match total cell (DAPI^+^ object) count: 7889 ± 342 from three linear 3D cultures and 22 ± 6% astrocytes in three 2D cultures) in a 4000 μm × 200 μm confinement with a height of 100 μm and 1600 randomly placed fixed surface anchoring points on culture substrate (Fig. 6d, top). We used four types of attraction forces in our model: (1) neuron-to-neuron (N-N), (2) neuron-to-astrocyte (N-A), (3) astrocyte-to-neuron (A-N), and (4) astrocyte-to-astrocyte (A-A). These forces where characterized by constants that determined their relative strengths. Repulsion strength *R* between cells was the same for all cell types. Since cellular aggregation halts after a certain period of time (Supplementary Video 1), we also introduced a cell movement freedom parameter. Cell movement freedom is set at 1 at the beginning, allowing free movement of the cells. After a certain period of time (after 50% of total simulation time in our simulation), this parameter drops in an inverse sigmoidal manner blocking cell movement (Fig. 6e). After the simulation, neurons aggregated into clusters with astrocytes concentrated at the surface (Fig. 6d, bottom). This result was strikingly similar to the observed morphology of linear 3D cultures (Fig. 2a, b). As hypothesized, we observed that the peak of the CCDP of simulated culture falls at the distance at which the cellular attraction and repulsion forces nullify each other. We named this point the force equilibrium point. To validate this finding, we systematically changed the force equilibrium point by changing the repulsion strength *R* (Fig. 6f). Increase in *R* shifted the force equilibrium point to the right, representing a larger characteristic cell-to-cell distance (Fig. 6f, inset). With all other parameters unchanged, we re-simulated the model with different values of *R*. Figure 6g shows the mean CCDPs of the resulting simulated cultures. Clusters did not form for total force equilibrium point at 50 and 60 μm and the CCDP for whole culture was calculated in these cases. We found a linear relationship between the peak of CCDP and the force equilibrium point (Pearson’s correlation coefficient, *r*^2^ = 0.96 between data points and fitted linear model in Fig. 6h).

We then optimized the parameters (parameter values are listed in “Methods”) to match the in vitro contraction, cluster number, and CCDP (Supplementary Fig. 2). The simulation provided a clear visual representation of cellular aggregation (Supplementary Video 2) and yielded contraction and the number of clusters within one std of the experimental values and a near identical CCDP. Pearson correlation coefficient between the experimentally obtained mean CCDP (Fig. 2c) and mean CCDP of three simulated cultures was *r* = 0.99 with *p* = 5.3e−117 (Supplementary Fig. 2e).

### Superficial astrocytic layer depends primarily on neuron to neuron and astrocyte to neuron adhesion

**Fig. 7:**
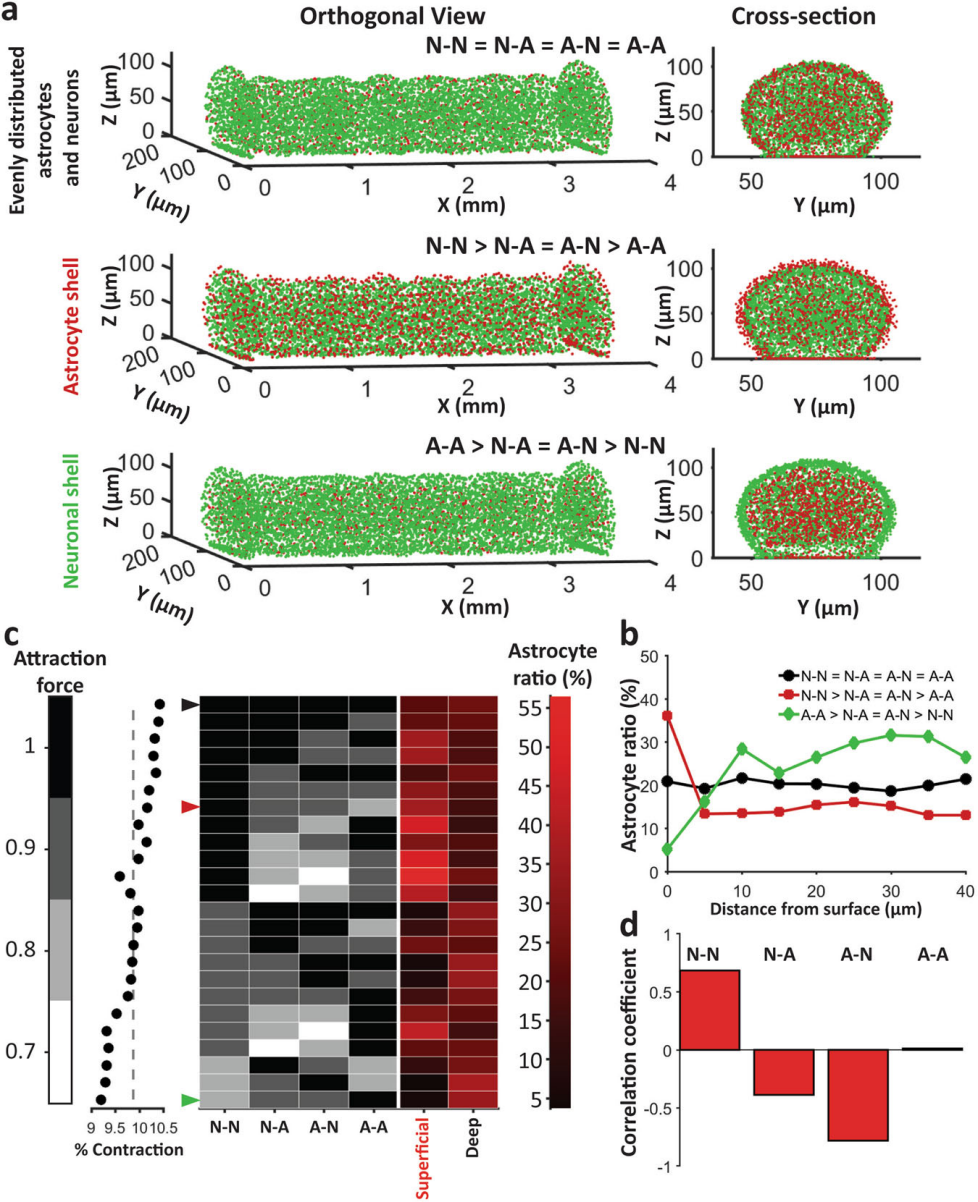
Simulated outcome of different attraction force configurations. **a** Orthogonal view (left) and cross section view (right) of three sample configurations: top: N-N = N-A = A-N = A-A configuration yielded uniformly distributed neurons and astrocytes, middle: N-N > N-A = A-N > A-A yielded astrocyte shell, bottom: A-A > A-N > N-A > N-N yielded neuronal shell. **b** Mean astrocyte to total cell ratio at different distance from surface for the three configurations in **a**. **c** % contraction (left, dot plot), Astrocyte ratio (right: shades of red) at surface and maximum astrocyte ratio in deep layers (more than 10 μm below surface) for different relative cell attraction constants (indicated by shades of black). Arrows indicate the cases shown in **a** and **b**. **d** Correlation coefficients between values of different adhesive forces and astrocyte ratio in the superficial layer.

Initially, we chose N-N > N-A > A-N > A-A as relative cell attraction force constants for our simulation. Now, we altered this order to observe the effect of neuron–astrocyte inter and intra relative attraction forces on cell aggregation and sorting in silico. Relative force order of N-N > N-A = A-N > A-A resulted in an astrocyte shell (Fig. 7a, middle, Fig. 7b, red trace), while force order of A-A > A-N = N-A > N-N resulted in an astrocyte-void neuronal shell (Fig. 7a, bottom, Fig. 7b, green trace). Keeping all the forces equal resulted in a uniform distribution of astrocytes (Fig. 7a, top, Fig. 7b, black trace). We then simulated 24 different force configurations shown in Fig. 7c. All of these configurations yielded equal number of clusters and similar contraction (within 1.5%, Fig. 7c, dot plot). The black arrow at the first row indicates the case of equal relative forces which yields uniform astrocyte distribution (same case is shown in Fig. 7a top, and by black trace in Fig. 7b). Red arrow (seventh row) indicates N-N > N-A = A-N > A-A case shown in Fig. 7a middle, and described by red trace in Fig. 7b. Green arrow (last row) shows A-A > N-A = A-N > N-N case corresponding to Fig. 7a bottom, and green trace in Fig. 7b. We observed that the existence of the astrocytic shell was heavily dependent on the relative order of forces. To analyze the relation between these forces and astrocytic shell and center cluster formation, we calculated cross-correlation coefficient of each force (force column, each of the gray column in Fig. 7c) and superficial astrocyte ratio (first red column in Fig. 7c). Neuron to neuron attraction force showed highest positive correlation (0.68) with superficial astrocyte ratio while astrocyte to neuron attraction force showed highest negative correlation (−0.78) with the same (Fig. 7d). Neuron to astrocyte attraction force also showed negative correlation (−0.38). Astrocyte–astrocyte attraction showed the smallest correlation (0.013).

### Astrocytes reduce contraction

**Fig. 8:**
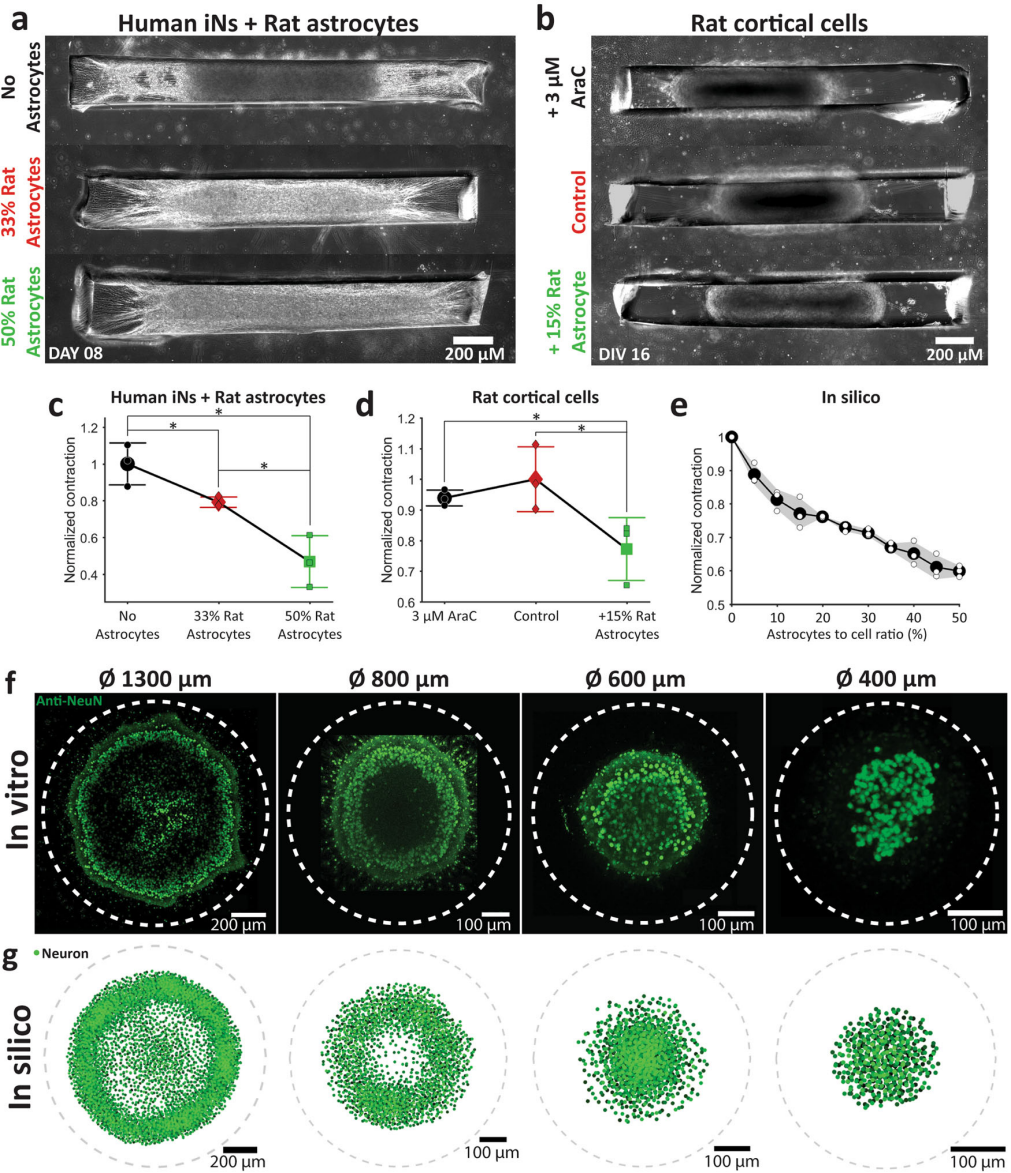
In silico model validation. **a** Human iNs cultures with top: no astrocyte, middle: 33% rat astrocytes and bottom: 50% rat astrocytes added. **b** Rat cultures top: chronically cultured in 3 μM AraC, middle: control, bottom: +15% astrocyte added. Mean with standard deviation of normalized contraction for different conditions in **c** Human iNs + rat astrocyte cultures, **d** Rat cortical cell cultures, and **e** in silico culture. Normalization was done first, with respect to culture size on the day 0 and then with respect to **c** mean of 0% astrocyte culture, **d** mean of control, **e** mean of 0% astrocyte culture contraction. Three cultures were analyzed for each condition of each type of culture. Asterisks indicate results of two-sample Kolmogorov–Smirnov test (*p < 0.05, **p < 0.01, and ***p < 0.001). **f** Projection of brightest points of all the confocal *z*-stacks of Anti-NeuN staining of (from left to right) 1300, 800, 600, and 400 μm diameter rat cortical cell cultures. Dashed lines indicate confinement. **g** In silico simulation results for confinement diameter (from left to right) 1300, 800, 600, and 400 μm.

To experimentally validate the relative roles of neurons and astrocytes found in our silico model, we created cultures in which the number of astrocytes could be precisely controlled. We differentiated human iPSCs by overexpression of NGN2 by lentiviral transduction^33^ to create induced neurons (iN). With puromycin purification, this method provided a rapid way to create highly pure neuronal population. We created three types of cultures: (1) 100% iNs, (2) 67% iNs + 33% rat astrocytes, and (3) 50% iNs + 50% rat astrocytes (Fig. 8a). These cultures exhibited similar morphology to rat cortical linear 3D cultures when observed by phase contrast microscope (Fig. 8a). In total, 100% iN culture showed maximum contraction whereas increase in astrocyte proportion resulted in a significant decrease in contraction (Fig. 8c). This suggests that contraction of 3D cultures is a neuron-driven process that is modulated by the presence of astrocytes. We then analyzed contraction in three types of rat 3D linear cultures: (1) control cultures from dissociated rat cortical cells (containing neurons and glia), (2) cultures from dissociated rat cortical cells maintained in the presence of 3 μM cytosine arabinofuranoside (AraC), and (3) cultures from 85% dissociated rat cortical cell +15% rat astrocytes (Fig. 8b). The motivation was to decrease astrocyte proportion in culture by mitotic inhibition with AraC or to increase astrocyte proportion by addition of extra rat astrocytes. Though AraC did not reliably increase contraction, +15% astrocyte culture showed a significant decrease in contraction (Fig. 8d). With N-N > N-A = A-N > A-A attraction force configuration, three sets of simulations of our in silico model with random initial cell placements and different astrocyte to cell ratio also replicated negative modulation of contraction by increased proportion of astrocytes (Fig. 8e). This validated our model’s ability to predict culture contraction based on cellular composition.

### In silico model recreates self-assembly of ring-like neuronal layers

Previously, we reported rat cortical 3D cultures in circular confinement^15,34^. These cultures showed significant change in morphology with change in confinement diameter (1300, 800, 600, and 400 μm, Fig. 8f). The most notable feature was the ring-like neuronal layer that was present in cultures in >800 μm diameter confinement, but not smaller confinements (Fig. 8f). There was also the formation of a dense neuronal cluster at the center of cultures in 1300 μm confinement, which was absent in cultures in 800 μm confinement (Fig. 8f). Here, we used the optimized in silico model described above to determine if it predicts effects of circular confinement. At first, we recreated experimental cell seeding by randomly placing 80% neurons and 20% astrocytes (at an in vitro-like^15,21^ concentration of 1 × 10^−4^ cells/μm^3^ with 10 μm minimum cell to cell distance) in a disk confinement of 1300, 800, 600, or 400 μm diameters with surface anchoring points spread randomly on culture substrate at a concentration 2 × 10^−3^ points/μm^2^ and a minimum 10 μm point to point distance. Without changing any other parameters in the previously optimized model, we then simulated these cultures. These simulations resulted in remarkably accurate morphology for all confinement diameters (Fig. 8g). This further validated our model’s ability to predict overall morphology of cultures with different confinement shapes.

## Discussion

In summary, we presented a culturing method that yielded confined and adhered linear 3D neuronal cultures that were characterized by the presence of neuronal clusters, a superficial astrocyte-rich layer, and aligned neuronal and glial processes in the central, but not lateral locations. We found that culture contraction depended mainly on N-N interactions and was inhibited by increasing the proportion of astrocytes. Lateral culture locations were rich in astrocytes and were characterized by a higher baseline [Ca^2+^] level. Neurons fired spontaneous synchronized bursts that initiated in culture centers but had higher dynamic [Ca^2+^] peaks in the lateral (edge) locations. We developed a computational model that could explain neuron and astrocyte aggregation, clustering, and sorting in this experimental model; and that was capable of correctly predicting morphology of confined cultures with different geometries.

Our in silico model is predicated on the existence of attractive and repulsive forces that govern collective cell movements in neuronal 3D cultures. These forces may be a combination of cell–cell interactions, cell–ECM interactions, chemotaxis, and other processes^35^. Both neurons and astrocytes express cadherins and integrins, which may mediate cell–cell and cell–ECM interactions, respectively^36–38^. Neurons and astrocytes are also capable of secreting extracellular matrix components that may interact with neighboring cells^39,40^. Both cell types also release signaling molecules, gradients of which may direct cell migration. The presence of dense neuropil (mixed axons, dendrites, and synapses that make up most of the space in the gray matter not occupied by cell bodies^28^) may exert a repulsive force on the cells, and may be the reason for the minimum distance between cell soma in this preparation. The presence of axons and dendrites makes neuronal 3D cultures qualitatively different from previously published experimental models of cell aggregation and sorting. These models were typically based on cell types that formed close-packed aggregates with cell-to-cell distances (center-to-center) between 10 and 30 µm^41,42^. Cell-to-cell interactions in these non-neuronal systems occur at somatic boundaries and are traditionally modeled by lattice-based computational models where cells are closely packed and occupy well-defined regions in space^43,44^. In contrast, neuronal dendritic arbors and branched axons are large compared to soma size, extending for hundreds of micrometers in both the intact cortex and in cultures (schematically illustrated in Fig. 6a). Direct N–N interactions (such as cadherin-mediated cellular adhesion) can thus occur at significantly longer distances than for other cells. This may explain the relatively long range of both attractive and repulsive forces compared to the diameter of neuronal soma. Another source of long-range forces may be cell–ECM–cell interactions. As mentioned above, neurons and astrocytes secrete ECM components, and ECM may be present in the 3D neuronal cultures. The relative roles of cell–cell interactions through dendrites and axons versus through ECM would be interesting to investigate in future studies, although the intricate geometry of dendritic arbors and axonal branches may create additional challenges. This complexity of interactions in neuronal-astrocyte cultures led us to construct a phenomenological in silico model that is based on total cellular attraction and repulsion. Our model does not assume the presence of specific modes or mechanisms of cell interaction. Nevertheless, it is capable of predicting the morphology of neuronal 3D cultures in different confinement geometries, including self-assembly of neuronal layers (Fig. 8f, g), and formation of neuronal clusters (Figs. 6d, 7a, and Supplementary Video 2). This in silico model may serve as a design tool for development of future neuronal 3D cultures intended for transplantation or for in vitro disease modeling.

We observed formation of an astrocyte-rich sheath around 3D cultures, similar to formation of a glial scar (layer rich in reactive astrocytes with elevated GFAP expression) around injury sites in the brain^8^. The 3D culture can be thought of as an inside-out model of response to injury: its interior region, rich with neurons and neuropil, is representative of brain parenchyma, while the exterior, astrocyte-rich region at the interface of parenchyma and culture medium can be thought of as the glial scar. Formation of the glial scar is thought to occur due to inflammation or wound healing like processes, where glial cells migrate to or proliferate at the site of injury due to activation of intracellular signaling^8–10,36^. One of the unexpected results of our in silico modeling was that the astrocyte sheath enveloping the 3D cultures may form due to different neuron and astrocyte attraction forces, rather than astrocyte signaling. Our results suggest that neurons drive aggregation due to their strong attraction forces, while astrocytes are left behind due to their relatively lower attraction forces. If the relative magnitudes of the attraction forces are reversed, a neuron-rich sheath forms instead in silico (Fig. 7). Reduced contraction in 3D cultures with increased number of astrocytes provides experimental support for this hypothesis. Both human and rat cultures with the lowest proportion of astrocytes showed the strongest contraction (Fig. 8a–d), suggesting that neurons have a stronger attraction drive. It is interesting to note that the word “glia”, describing astrocytes as well as oligodendrocytes, comes from the Greek word “glue”, reflecting the old presumption that these cells hold the central nervous tissue together. Our results suggest that it is actually neurons that “glue” (i.e., strongly attract) dissociated cells together leading to 3D aggregation followed by contraction in linear 3D cultures. It is interesting to compare culture contraction to the phenomenon of “compaction” described in spheroids of progenitor cells^45^ and in developing embryos^46–49^. Cell-to-cell adhesion and tension are hypothesized to be the driving forces behind compaction, which occurs due to cells losing their spherical shape and maximizing the surface area of cell-to-cell contacts. Neurons and astrocytes are also spherical immediately after dissociation and seeding into the PDMS slit; thus, initial culture contraction likely occurs due to these cells losing their spherical shape and maximizing surface in contact with other cells, with neurons providing the dominant force as described above. Contraction at later time points, however, likely involves “compaction” not just of cell bodies, but also of actively growing neuronal and astrocytic processes, and would be an interesting topic of future investigation.

As noted above, our results suggest an alternative hypothesis for glial scar formation: astrocyte-rich layer at the site of injury may occur due to collective migration of neurons away from the injury due to strong neuron–neuron attraction forces. We noted that, in addition to a higher proportion of astrocytes in the superficial layers, astrocytic processes expressed more GFAP in superficial compared to deep culture layers (Fig. 3g, h). This is indicative of reactive nature of superficial astrocytes in this model system, suggesting that inflammatory processes are occurring in parallel with cell aggregation. Our culture system may prove to be a useful in vitro model for studies aimed at understanding glial scar formation in traumatic brain injuries and spinal cord injuries.

We found that astrocytes have a strong positive effect on neuronal activity (Fig. 4a–d), which is consistent with the previously established neuron–astrocyte interactions^50–52^. Interestingly, neuronal activity was culture location-specific (edges vs. center), in terms of baseline and dynamic [Ca^2+^] intensity and burst initiation (Figs. 4e, h and 5). We found that the morphology of the culture in the center and at edges was not the same, with more neuronal and astrocytic processes aligned at the center compared to the edges (Fig. 3a–f). These differences in alignment may be due to different physical forces and migration paths experienced by cells in different locations during the course of aggregation. This may also be due to preferential growth of neuronal axons and dendrites along the long axis of the linear 3D culture. Correlation of differences in neuronal activity with morphology suggests that there is a structure–function relationship in these neuronal constructs. Neurite alignment, particularly that of apical dendrites, is a prominent feature of the cortex^53^, and may play a role in spontaneous activity generation in the linear 3D cultures. This underscores the need for understanding aggregation and sorting when developing neuronal constructs for implantation or for in vitro disease modeling. Brain injury and brain gliomas are associated with abnormal neuronal activity, including seizures^54–56^. Our results suggest that tissue discontinuity and boundaries, which in our model are introduced by presence of the confining slit, and in the brain by injury or by presence of a tumor, may cause rearrangement of neurons, glia, and their processes, which in turn could lead to location-specific patterns of neural activity. Linear 3D cultures may thus find use in studies aimed at understanding glioma or injury-associated seizures.

Our technique for creating 3D aggregated neuronal culture uses poly-lysine coated culture substrate to which neurons and astrocytes readily attach. This is in contrast to more widely used low cell attachment surfaces for production of scaffold-free aggregated spheroid cultures^11,57^. We found that the presence of a cell-adhesive substrate enables creation of non-spheroid aggregate shapes, such as the linear 3D culture described in this work. Without poly-lysine coating, we found that neurons and astrocytes aggregate faster and the construct escapes PDMS slit confinement and turns into a spheroid. This is undesirable because spheroids with diameter exceeding a few hundred micrometers suffer from necrosis due to limited oxygen and nutrient supply at the spheroid core. Because of the linear 3D cultures’ adherence to surface and low height, they do not undergo necrosis. Development of a predictive in silico model of neuronal aggregation required accurate quantification of culture contraction and cluster formation, both made possible by the long (up to 4 mm) dimension of the adhered linear 3D cultures.

## Methods

### PDMS confinement device

A 4-inch silicon wafer was spin coated (600RPM for 90 s) with liquid PDMS and cure mixture (1:10) (Sylgard 184 by Dow Corning). After overnight baking, a 100 μm thick PDMS film was obtained. PDMS film was then removed from silicon wafer and placed on a clean plastic 150 mm petri dish. We printed (4 mm × 200 μm or 2 mm × 200 μm) rectangles on white paper and attached it below the outside of 150 mm petri dish. Slits in the 100 μm high PDMS film were then manually cut with a scalpel following the printed rectangles. Scalpel was held at a right angle to make non-oblique cuts. The device was then cut out and submerged in a container with 200 proof ethanol for 5 min in a sterile biosafety hood. The device was then air dried completely in sterile environment. Plastic or glass plasma treated cell culture surface was then covered with filtered (0.22 μm vacuum filter, Millipore Sigma, Catalog # SCGP00525) 1 mg/ml Poly-D-Lysine solution (Sigma-Aldrich, Cat # P6407-5MG) in 0.1 M borate buffer (pH 8.5) overnight. The substrate was then washed thrice with dH_2_O (2 × washes + 1 30-min wash) and dried completely. Clean and dry PDMS device was then placed on this dry Poly-D-Lysine coated substrate. A sacrificial layer with same slit dimensions was stacked on this device (Fig. 1) to remove out-of-confinement cells. The whole device was then submerged in culture medium and incubated overnight to minimize the risk of PDMS toxicity. Prior to cell seeding, any bubbles stuck in the slit were removed by blowing culture medium in the submerged slits with a pipettor. The submerged devices were then placed in vacuum desiccator for 30 min and the remnant bubbles were again removed by blowing culture medium into the submerged slit. This desiccator-blow process was repeated as needed. Removing bubbles was important for spreading cell suspension uniformly across the whole slit. We observed that cells could not be seeded in the area of the slit where a bubble previously existed. The culture medium was then removed, and the top part of the sacrificial device was dried out with a vacuum aspirator pipet. Dry top surface helped to maintain the shape of initially seeded cell droplet (Fig. 1, 1st row, 3rd image). If the surface was left wet, cohesive force of the remnant culture medium pulled the initially seeded droplet away from the slit.

### Rat dissociated cortical cells

We obtained cortices from neonatal rats (postnatal days 0–1 Sprague–Dawley rat pups, Charles River Laboratories) according to the protocol developed by Brewer et al.^58^. Papain (PDS Kit, Papain Vial, Worthington Biochemical Corporation) dissociation system was used for dissociation. All animal use protocols were approved by the Institution Animal Care and Use Committee at Lehigh University and were conducted in accordance with the United States Public Health Service Policy on Humane Care and Use of Laboratory Animals. Obtained cells were centrifuged at 800 RPM for 5 min and appropriate amount of culture medium was added to obtain 40 million cells/ml concentration.

### Rat astrocytes

One million rat cortical cells obtained as described above were plated on PDL-coated cell culture treated T75 flasks (with 13 ml culture medium). Cells were cultured in 10% fetal bovine serum (FBS) (Invitrogen, Cat# 26140087) in Neurobasal-A (Invitrogen, Cat# 10888022) supplemented with 0.3% 30 µg/ml gentamicin (Life Technologies). Full culture medium was replaced every other day. Cells were used for experiments or cryopreserved for future use after at least one passage to reduce the percentage of neurons.

### Linear 3D cultures from rat cells

Cell suspension with increased percentage of astrocytes was created by mixing desired volume of cortical and astrocyte (both 40 million cells/ml) suspensions. In total, 2 μl droplet of cell suspension was then seeded on the PDMS device on PDL-coated surface at the edge of the slit (Fig. 1, 1st row, 3rd image). Cells do not enter the slit spontaneously due to the cohesive force of the cell suspension. However, a light nudge with fine-tip-forceps forces the cell suspension to get in the slit and the capillary force then pulls and distributes the cell suspension evenly throughout the confinement (Fig. 1, 1st row 4th, and 2nd row 1st image). Another 2 μl cell suspension was added after the device was populated. The whole device was then submerged carefully in the seeding medium (10% FBS (Invitrogen, Cat# 26140087) in Neurobasal-A (Invitrogen, Cat# 10888022) supplemented with 0.25% 0.5 mmol/l GlutaMAX (Life Technologies) and 0.3% 30 μg/ml gentamicin (Life Technologies)). Medium was added to the side of the petri dish, and the petri dish was then gently tilted to submerge the device with minimal disturbance to the seeded cells. The sacrificial layer was removed after 1 h when the cells have settled down. After removing the sacrificial layer, initial seeding medium was aspirated gently from the side of the petri dish. The devices were then submerged again in fresh seeding medium and incubated at 37 °C and 5% CO_2_ for 1 h. After 1 h, seeding medium was removed and culture medium (2% B27 supplement in Neurobasal-A supplemented with 0.25% 0.5 mmol/l GlutaMAX and 0.3% 30 μg/ml gentamicin) with (1:1000 dilution) adeno-associated virus particles containing genetically encoded calcium indicator jRGECO1a^31^ construct under Syn promoter (100 µl at titer ≥1 × 10¹³ vg/ml) was added. The culture was then incubated at 37 °C and 5% CO_2_. Half of culture medium was replaced with fresh culture medium every 3 days.

### Linear 3D culture from hiPSC derived iNs and rat astrocytes

Human-induced pluripotent stem cells (hiPSCs) were purchased from WiCell (CBiPS-LZ6-2-PCBC). Functional human iNs were differentiated from these hiPSCs by overexpressing neurogenin-2 (Ngn2)^33^ using the modified protocol developed by Zhang et al.^59^. The required plasmids to create lentivirus were: pMD2.G (Gift from Didier Trono (Addgene plasmid # 12259; http://n2t.net/addgene:12259; RRID:Addgene_12259)), pRSV-rev^60^ (Gift from Didier Trono (Addgene plasmid # 12253; http://n2t.net/addgene:12253; RRID:Addgene_12253)), pMDLg/pRRE^60^ (Gift from Didier Trono (Addgene plasmid # 12251; http://n2t.net/addgene:12251; RRID:Addgene_12251)), FUW-M2rtTA^61^ (Gift from Rudolf Jaenisch (Addgene plasmid # 20342; http://n2t.net/addgene:20342; RRID:Addgene_20342)), pTet-O-Ngn2-puromycin^33^ (Gift from Marius Wernig (Addgene plasmid # 52047; http://n2t.net/addgene:52047; RRID:Addgene_52047)), and positive control: FUW-Tet-O-EGFP^62^ (Gift from Stefano Piccolo (Addgene plasmid # 84041; http://n2t.net/addgene:84041; RRID:Addgene_84041)).

We adopted and modified feeder-free differentiation described in the protocol^59^ and used rat astrocytes instead of mouse glial cells. This protocol provided a convenient and rapid way to differentiate iNs by doxycycline conditioned overexpression of NGN2 and puromycin based purification. Rat astrocytes were obtained by following the protocol described above in “Rat astrocytes” section. After puromycin purification, iNs were dissociated using Accutase (Stemcell Technologies, Catalog# 07920) and counted. Desired amount of medium was added to create 40 million iNs/ml suspension. Appropriate volume of astrocytic cell suspension with same cell concentration and medium was mixed with this iN suspension to create seeding cell suspension. Cells were seeded following the protocol described in the previous section. Neurobasal Medium + 1% L-Glutamine (200 mM) + 2% ml B27 Supplement (50x) + 5000x BDNF (50 μg/ml) +5000x NT-3(50 μg/ml) + 5000x Laminin(1 mg/ml) + 1000x Doxycycline (2 mg/ml) + 2 μM Cytosine β-D-arabinofuranoside hydrochloride (AraC) were initially used in these cell suspensions and to seed cells. Half medium was replaced with fresh medium every 3 days until 7-day post-seeding. After 7th day, half of the medium was replaced with 2-AraC Neuronal growth medium (90% MEM + 2.5% of 20% w/v glucose + 0.25% of 8% w/v NaHCO_3_ + 0.1 mg/ml transferrin + 5% FBS + 2% B27 Supplement (50x) + 0.25% 200 mM L-glutamine + 0.05% 4 mM AraC). In total, 500 μl of culture medium was removed and 600 μl of 2-AraC Neuronal growth medium was added every 7th day after 10 days post-seeding.

### Immunohistochemistry

Cultures were fixed in 4% paraformaldehyde on DIV 14 for 1 h. Cell permeabilization was done with Triton X-100 (Sigma-Aldrich) in PBS for 2 h on a shaking platform. Cultures were then blocked using 10% goat serum in PBS for 1 h. Antibodies (Anti-GFAP antibody (Abcam, ab7260) conjugated to Alexa Fluor 647(Thermo Fisher, A27040) and Anti-NeuN (Millipore Sigma, MAB377) or Anti-β-3-Tubulin (Thermo Fisher Scientific, MA1-118) conjugated to Alexa Fluor 488 (Thermo Fisher Scientific, A28175) were then applied to the cultures on a shaker at +4 °C for 48 h. Cultures were counterstained with DAPI. A drop of Fluoro-Gel (Electron Microscopy Sciences, Catalog # 17985-10) was added on the fixed and stained culture. Spacer glass coverslips were placed at two sides of the culture and another coverslip was added on top of the culture to create a sandwich. Cultures were then imaged using a confocal microscope (Zeiss LSM 510 META, Germany) with ×40 objective. Distance between optical slices was 1 μm, and cultures were imaged over their entire depth. Images were then processed in Fiji (ImageJ).

### Cell to cell distance profile (CCDP)

CCDP for a ROI or a cluster was calculated from all counted cell to cell distance for that ROI or a cluster (more details in next paragraph). At first, 5-μm bin width histogram was created from these calculated distance values. Each bin count was then divided by total distance count (area of total histogram) to obtain probability normalized histogram. We termed this probability normalized cell to cell distance histogram as CCDP.

For Nissl stained rat brain section from BrainMaps^26^, neurons were counted by identifying their cellular shape manually^63^. In total, 200 μm × 200 μm ROI were chosen from a *z*-plane within a cortical layer with homogeneous cell distribution. The centers of these neurons were marked in *X*–*Y* plane and distances (in *X*–*Y* plane) between all centers for a ROI was calculated and used to calculate CCDP for that ROI.

In linear 3D cultures, NeuN^+^ cells were counted using 3D Watershed in Fiji (ImageJ) and the center of mass of each cell was detected. Number of neurons in each 10 μm bin along *x-*axis (along culture length) was then counted. Local minima positions of this cell count vs *x*-position then indicated individual cluster edges. Culture edges were detected by using a threshold. The whole culture was divided into 10 μm bins along *z*-axis. Distances between cell centers for each bin in a cluster (between two cluster edges or culture edge and cluster edge for first and last clusters) were calculated separately for all cells in that cluster in MATLAB. Distances counted from all the *z*-bins for a cluster were then used to calculate CCDP for that cluster.

### Processes alignment detection

Z-stacks of ×40 confocal images were divided into 10 μm × 10 μm ROI, and the ROIs were then thresholded (second row of Supplementary Fig. 1, threshold grayscale value: 55). 2D correlation coefficient was then calculated for each pixel displacement (Supplementary Fig. 1, third row from top). This provided a clearly distinguishable result for ROIs with visible processes alignment (first and third columns of Supplementary Fig. 1) and no alignment (second and last columns of Supplementary Fig. 1). If both thresholded binary images have *M* × *N* pixels, then the 2D cross-correlation image would be (2 × *M* − 1) by (2 × *M* − 1) pixel image, with each pixel value defined as:

1 $$C\left(k,l\right)=\mathop{\sum }\limits_{m=0}^{M-1}\mathop{\sum }\limits_{n=0}^{M-1}X(m,n)\times Y(m-k,n-l)$$

$${\rm{where}},-\left(M-1\right)\le k\le \left(M-1\right)\;{\rm{{and}}}-\left(M-1\right)\le l\le (M-1)$$

This 2D correlation image was again thresholded at 70% of image maximum value. Aligned ROIs provided skewed ellipsoids and un-aligned ROIs provided almost circular shape after this step. Major and minor axes of the structure at the center of thresholded 2D cross-correlation images were then calculated. The axis detection was performed using following algorithms:

1. Edge points detection.
2. Find maximum distance between the edge points: major axis.
3. Minor axis was detected by finding the line that goes through the middle of the major axis and has shortest distance.

If the ratio of major and minor axis was <2, or the length of major axis was <5 pixels, the ROI is marked as non-directional. Otherwise, the ROI was marked to have an alignment (directional ROI) and the angle of major axis with *x*-axis (long axis of the linear 3D culture) was calculated as the alignment angle of that ROI.

### Nucleus alignment and height detection

We manually outlined the nucleus bounding 3D cuboid ROI in confocal *z*-stacks in FIJI ImageJ. All of the nuclei were of ellipsoid shape. Each nucleus’ major axis (alignment direction) and height was then detected from these 3D ROIs (cuboids) separately using MATLAB by following the algorithm below:

1. The cell border was cleared in a 3D ROI containing one nucleus by suppressing light structures at the border (MATLAB function: imclearborder() with six pixel connectivity).
2. The edge was then smoothed by erosion at each *z*-plane of the 3D ROI. (MATLAB function: imerode()).
3. The cell outline was detected at each *z*-plane. (MATLAB function: bwperim()).
4. Distances (3D, Euclidean) between all edge points were detected.
5. Points separated by maximum distance were detected. The connecting line of these two points was considered to be the major axis of the ellipsoid nucleus.
6. The angle of the line in *X*–*Y* plane with *X*-axis (culture long axis) was then counted as that nucleus’ alignment direction.
7. Cell height was calculated from the distance (along *z*-axis) between highest and lowest edge points.

### Calcium imaging and analysis

Optical recordings of intracellular [Ca^2+^] indicated by jRGECO1a fluorescence were performed by placing cultures in a mini incubator (Bioscience Tools) kept at 37 °C with a constant supply of humidified blood gas (5% CO_2_, 21% oxygen, balanced nitrogen, airgas) on a stage of a fluorescent inverted microscope (Olympus). Changes in fluorescence were observed via ×4 objective at 60 ms exposure and camera frame rate of 200 ms/frame. Optically recorded data were analyzed using ImageJ and MATLAB.

ROI were drawn either around the whole visible culture or portions of interest. Mean fluorescence in that ROI was calculated for each time point. The baseline was then determined using algorithm developed by Eilers and Boelens, 2005 for each time point. Mean fluorescence was then converted to Δ*F*/*F*. If raw gray value data is *R* and baseline is *F*_0_, then:

2 $$\frac{\triangle F}{F}=\frac{R-{F}_{0}}{{F}_{0}}$$

Activity was then detected by thresholding (3 × std) *ΔF/F* values. Cultures were recorded on DIV 10, 12, 14, and 16.

### Multi-electrode array (MEA) recording and analysis

Extracellular electrical recordings were performed using MEA with 60 round titanium nitride (TiN) electrodes of 30 μm diameter with glass ring and O-ring (60MEA 200/30 IR-TI, Multichannel systems). Clean, sterile, and dry PDMS devices were attached on these MEAs so that two electrodes were located under the edge of the culture and two electrodes under the middle of the culture. The PDMS devices formed a stable water-tight seal with the glass substrate of MEA. The internal reference electrode was initially covered with a plain PDMS film of appropriate size so that cells could not grow onto the reference electrode. The film was removed on DIV 12 prior to recording. Recordings were performed in a mini incubator at 37 °C with constant supply of humidified blood gas. The extracellular field potential signals from electrodes were fed into an amplifier (RZ2, Tucker Davis Technologies) fitted with high-impedance multiple-channel preamplifier stage (PZ2-64, Tucker Davis Technologies). In total, 6 kHz sampling rate and gain of 1000 were used. OpenEx (Tucker Davis Technologies) and MATLAB were used for signal processing and data analysis. A 100 Hz–3 kHz band pass filter was first applied to the raw data. Digital band stop filters of 60 Hz and its harmonics up to 3 kHz was then used to denoise the data. Spikes were detected using an automated thresholding method^64^. Recordings were performed every other day from DIV 10 to DIV 18. Recording time length was 20 min with 15 min initial stabilization time.

Silent 100 ms part of each recording from each electrode was then manually selected and the std of this segment was used as threshold for activity detection for that electrode during that recording session. If a point (red filled circles in Fig. 5c, e) was over this threshold (red horizontal dashed line in Fig. 5c, e), it was counted as an initiation point if for the next 10 ms (from red filled circle to vertical cyan line in Fig. 5c, e), all points stayed above threshold. New points were not registered until trace went below threshold. Burst initiation times were extracted from center and edge electrode traces and delay was assessed for each burst from the differences between these two time points (from green to red solid vertical line Fig. 5c, e).

### In silico model

To model the self-assembly observed in linear 3D cultures, we represented each cell as a discrete object. At time = 0, simulation was initiated with random cell placement (at a concentration 1 × 10^−4^ cells/μm^3^ with minimum 10 μm distance between centers) within the confinement region (modeling PDMS slit). In total, 20% of these cells were randomly marked as astrocytes and the rest were marked as neurons (Fig. 6d, top, green-neuron, red-astrocytes). Surface adhesion was modeled by randomly distributed fixed cells on basal (substrate) surface (2 × 10^−3^ points/μm^2^, Fig. 6d, black dots on the surface). We opted to use three single exponential forces: (1) cell to cell attraction force, 2) cell to cell repulsion force (Fig. 6c), and (3) cell to surface adhesion force. Force length constants in all cases were defined by the length at which a force drops to its 1% of maximum of value. Each cell experienced an attraction, repulsion and surface adhesion force from all other cells and surface adhesion points.

The attraction force (*ax*) and repulsion force (*rx*) on cell *i* due to cell *j* in *x* direction were:

3 $$a{x}_{i,j}=A\times {e}^{{\frac{{\ln}(0.01)} {{\tau }_{a}}} \times ({x}_{j}-{x}_{i})}$$

4 $$r{x}_{i,j}=R\times {e}^{{\frac{{\ln}(0.01)}{{\tau }_{r}}} \times {x}_{i,j}}$$

The surface adhesion force (*sx*) between cell *i* and surface adhesion point *n* along *x* direction was:

5 $$s{x}_{i,n}=S\times {e}^{{\frac{{\ln}(0.01)}{{\tau }_{s}}} \times ({x}_{n}-{x}_{i})}$$

where *A*, *τ*_*a*_, and *R*, *τ*_*r*_ were attraction and repulsion strength and length constants, respectively. *S* and *τ*_*S*_ were surface adhesion strength and length constants. *x*_*i*_, *x*_*j*_, and *x*_*n*_ were *x* co-ordinates of cells *i*, *j*, and surface point *n*. For *N*_*C*_ total cells and *N*_*S*_ surface adhesion points, total force *Fx*_*i*_ experienced by a cell *i* along *x* direction was:

6 $${{Fx}}_{i}=\mathop{\sum }\limits_{j=1}^{{N}_{c}}{{AS}}_{i,j}\times {{ax}}_{i,j}-\mathop{\sum }\limits_{j=1}^{{N}_{C}}{{rx}}_{i,j}+\mathop{\sum }\limits_{n=1}^{{N}_{S}}{{sx}}_{i,n}$$

*AS*_*i*,*j*_ was a constant that was defined by type of interacting cells and could have four distinct values:

1. Attraction felt by a neuron due to another neuron (N-N, cells *i* and *j* were both neurons).
2. Attraction felt by a neuron due to astrocyte (N-A, cell *i* was a neuron, *j* was an astrocyte).
3. Attraction felt by an astrocyte due to neuron (A-N, cell *i* was an astrocyte, *j* was a neuron).
4. Attraction felt by an astrocyte due to another astrocyte (A-A, cells *i* and *j* were both astrocytes).

Forces along *y* and *z* directions (*Fy*_i_ and *Fz*_i_) were calculated similarly. The cellular displacement for one time step is then:

7 $$\triangle {x}_{i}=k\times {{Fx}}_{i}$$

8 $$\triangle {y}_{i}=k\times {{Fy}}_{i}$$

9 $$\triangle {z}_{i}=k\times {{Fz}}_{i}-g$$

where *g* is gravitational downward pull constant and *k* is the cell movement freedom constant (Fig. 6e). *k* = 1 at the beginning at the simulation. As culture matures, *k* decreases in a sigmoidal manner to replicate tissue “stiffening”.

10 $$k=1-{\frac{{e}^{\frac{{\rm{Simulation}}\; {\rm{end}}\; {\rm{time}}-{\rm{Stiffening}}\; {\rm{delay}}} {{\rm{Stiffenning}}\; {\rm{constant}}}}}{1+{e}^{\frac{{\rm{Simulation}}\; {\rm{end}}\; {\rm{time}}-{\rm{Stiffening}}\; {\rm{delay}}}{{\rm{Stiffenning}}\; {\rm{constant}}}}}}$$

Cells were not allowed to move outside confinement or below base (substrate). There was no boundary at the top.

Stiffening delay was introduced to allow cells to move freely at the beginning of the simulation, corresponding to the time at the beginning of the experiment when culture contraction is at its fastest.

The simulation parameters were initially chosen as follows: Total simulation time = 350, Stiffening delay = 300, Stiffening constant = 20, *A* = 1, *τ*_*a*_ = 320 μm, *R* = 2.27, *τ*_*r*_ = 140 μm, *S* = 0.01, *τ*_*s*_ = 30 μm, *g* = 1e−4, N-N = 1, N-A = 0.9, A-N = 0.8, and A-A = 0.7.

The optimized parameters were as follows: Total simulation time = 350, Stiffening delay = 300, Stiffening constant = 20, *A* = 1, *τ*_*a*_ = 320, *R* = 2.27, *τ*_*r*_ = 200, *S* = 0.01, *τ*_*s*_ = 30 μm, *g* = 1e−4, N-N = 1, N-A = 0.9, A-N = 0.8, and A-A = 0.7.

Model was implemented in MATLAB. Code and user guide are publicly available on Zenodo via GitHub^65^.

### Statistics and reproducibility

Two-sample Kolmogorov–Smirnov test was used for comparing two un-paired datasets. For paired datasets, paired-sample *t*-tests were performed. Statistical significance was calculated at a confidence level of 5% for both cases.

At least three cultures from two separate dissections were used to obtain data in each experiment. All source data underlying graphs in the main figures is available in Supplementary Data 1.

### Reporting summary

Further information on research design is available in the Nature Research Reporting Summary linked to this article.

## Supplementary information

Supplementary Information

Description of Additional Supplementary Files

Supplementary Video 1

Supplementary Video 2

Supplementary Data 1

Reporting Summary

## Data Availability

The datasets generated during and/or analyzed during the current study are available from the corresponding author on reasonable request. All source data underlying graphs in the main figures is available in Supplementary Data 1.

## Code availability

The in silico cellular aggregation model was implemented in MATLAB 2020b. A user friendly app of this model and user guide with step by step detailed instructions are publicly available on Zenodo via GitHub^65^. Optimized parameters obtained in this work are set as default in the fully editable fields in this app.
