# Supplementary material for: Neuron and astrocyte aggregation and sorting in three-dimensional neuronal constructs
Source: Commun Biol. 2021 May 17;4:587. doi: 10.1038/s42003-021-02104-2 (PMC8129100; doi:10.1038/s42003-021-02104-2)
Supplement: Supplementary file 1 — Supplementary Information [file 42003_2021_2104_MOESM1_ESM.pdf]

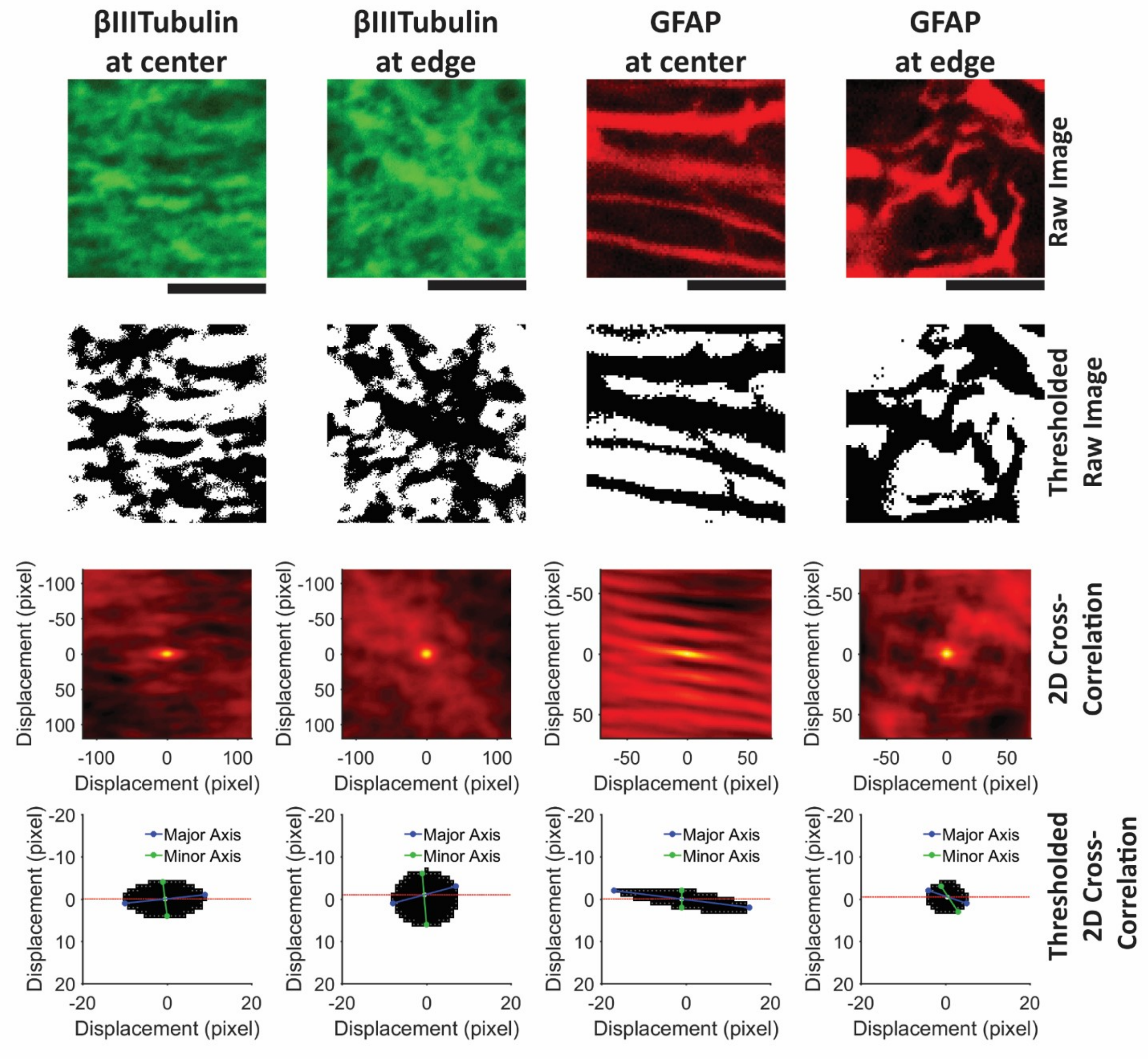

Supplementary Fig. 1| Processes alignment detection algorithm. Left two columns show analysis in Anti-βIII Tubulin and right 2 columns show analysis with Anti-GFAP. Top row shows raw image, Second from top row shows thresholded image. 3rd from top row shows 2D cross-correlation image. Bottom row shows thresholded image of 2D-cross-correlation (zoomed in). Red line indicates culture long edge, angle between red line and major axis (blue line) is considered as the angle of that region of interest if the ratio of length of major axis (blue line) and minor axis (green line) is greater than threshold (2). Scalebar: 5 µm for 1st and 2nd row from top.

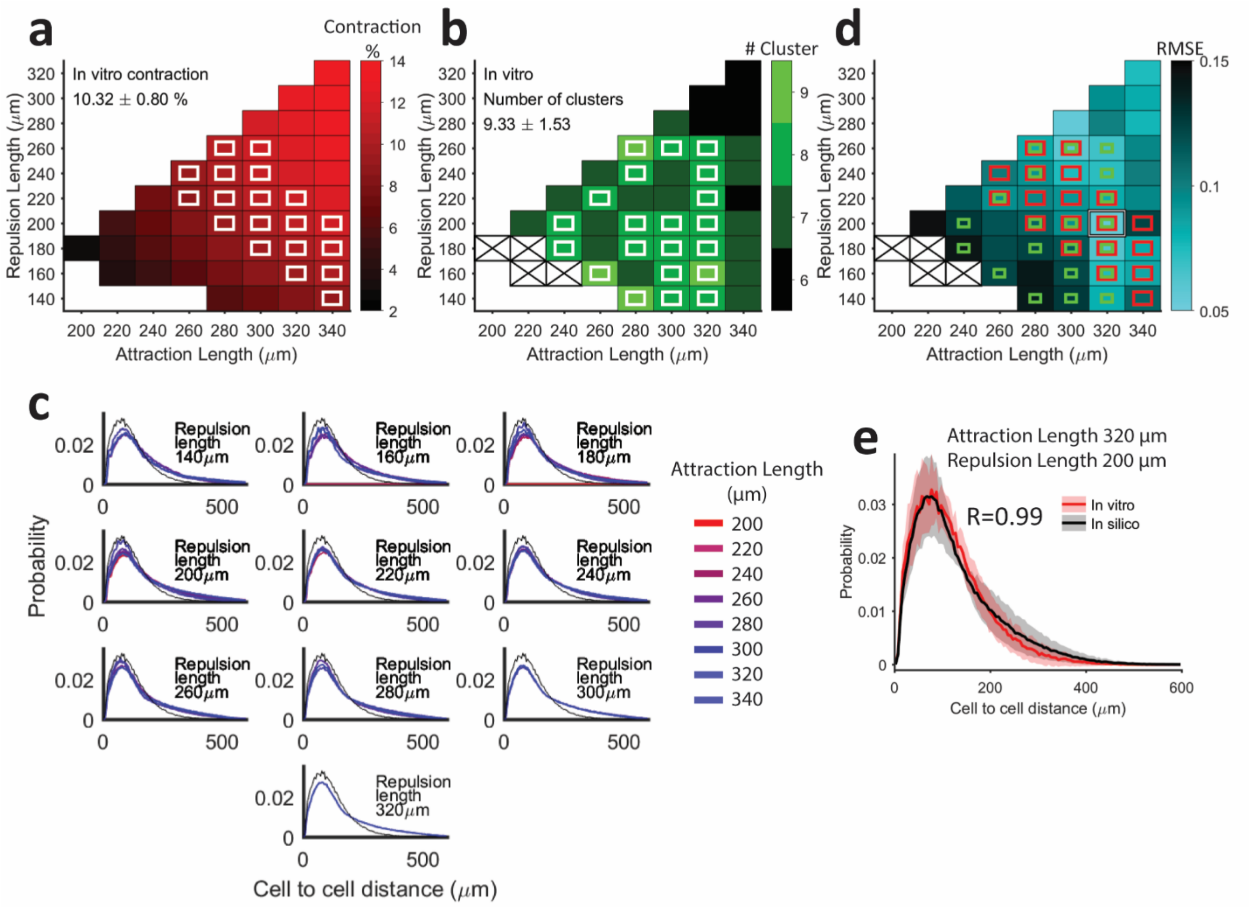

Supplementary Fig. 2| Model optimization. For different attraction and repulsion length, the in-silico **a,** % Contraction, **b,** Number of clusters is calculated and compared with in vitro values. (White rectangles indicate value within one standard deviation of corresponding in vitro value). **c,** CCPD is calculated for these scenarios. **d,** Root mean square error between mean in-silico CCPD and in vitro CCPD. Attraction and repulsion lengths that yield % contraction (white rectangles in **a**, red rectangle in **d**) and cluster number (white rectangles in **b**, green rectangle in **d**) within one standard deviation of in vitro mean is overlaid on **d**. Optimal value is detected from the values that were within range in both **a** & **b** (Green and red rectangles in **d**) and have the lowest RMSE value (Attraction length = 320. Repulsion length=200). **e**, mean (red trace) CCPD of 30 clusters from 3 (in vitro) linear 3D cultures (11, 8 and 11 clusters) with standard deviation (red shaded region) and mean (black trace) CCPD of 8 clusters from in silico final culture simulated with optimized model and standard deviation (black shaded region). Optimized model yields highly in vitro-like CCPD with a Pearson correlation-coefficient of 0.99.
