# Supplementary material for: Neuron and astrocyte aggregation and sorting in three-dimensional neuronal constructs
Source: Commun Biol. 2021 May 17;4:587. doi: 10.1038/s42003-021-02104-2 (PMC8129100; doi:10.1038/s42003-021-02104-2)
Supplement: Supplementary file 2 — Description of Additional Supplementary Files [file 42003_2021_2104_MOESM2_ESM.pdf]

Description of Additional Supplementary Files

File name: Supplementary Video 1

Description: TFormation of linear 3D culture. Left: Phase contrast images of dense dissociated rat cortical cell suspension seeded in 2 mm X 200 μm slit in 100 μm high PDMS device (DIV 01-18, one image per DIV) shows gradual formation of linear 3D culture over 8 days of incubation. **Right:** Length of 3D culture (from left image) on each DIV shows culture length stabilization after DIV 12.

File name: Supplementary Video 2

Description: Simulation video of optimized model. Top text indicates simulation time. **Top:** Side view from right angle to long edge, **Middle left:** Side view from right angle to short edge, **Bottom:** Top view, **Middle right:** 3D view. Green dots indicate neurons, Red dots indicate astrocytes, Cyan lines indicate confinement, there is no confinement at top. Black dots indicate fixed surface adhesion points.

File name: Supplementary Data 1

Description: All source data underlying graphs in the main figures.
