# Supplementary material for: Neuron and astrocyte aggregation and sorting in three-dimensional neuronal constructs
Source: Commun Biol. 2021 May 17;4:587. doi: 10.1038/s42003-021-02104-2 (PMC8129100; doi:10.1038/s42003-021-02104-2)
Supplement: Supplementary file 6 — Reporting Summary [file 42003_2021_2104_MOESM6_ESM.pdf]

nature research

Corresponding author(s): Yevgeny Berdichevsky
Last updated by author(s): Mar 24, 2021

Reporting Summary

Nature Research wishes to improve the reproducibility of the work that we publish. This form provides structure for consistency and transparency in reporting. For further information on Nature Research policies, see our Editorial Policies and the Editorial Policy Checklist.

Statistics

For all statistical analyses, confirm that the following items are present in the figure legend, table legend, main text, or Methods section.

n/a	Confirmed	
☐	☒	The exact sample size (n) for each experimental group/condition, given as a discrete number and unit of measurement
☐	☒	A statement on whether measurements were taken from distinct samples or whether the same sample was measured repeatedly
☐	☒	The statistical test(s) used AND whether they are one- or two-sided<br>*Only common tests should be described solely by name; describe more complex techniques in the Methods section.*
☐	☒	A description of all covariates tested
☐	☒	A description of any assumptions or corrections, such as tests of normality and adjustment for multiple comparisons
☐	☒	A full description of the statistical parameters including central tendency (e.g. means) or other basic estimates (e.g. regression coefficient) AND variation (e.g. standard deviation) or associated estimates of uncertainty (e.g. confidence intervals)
☐	☒	For null hypothesis testing, the test statistic (e.g. F, t, r) with confidence intervals, effect sizes, degrees of freedom and P value noted<br>*Give P values as exact values whenever suitable.*
☒	☐	For Bayesian analysis, information on the choice of priors and Markov chain Monte Carlo settings
☐	☒	For hierarchical and complex designs, identification of the appropriate level for tests and full reporting of outcomes
☐	☒	Estimates of effect sizes (e.g. Cohen's d, Pearson's r), indicating how they were calculated

Our web collection on statistics for biologists contains articles on many of the points above.

Software and code

Policy information about availability of computer code

Data collection	OptixCam OCView 6.3 was used for phase contrast imaging.<br>IC Capture 2.5 was used for recording Optogenetic Ca2+ indicator.<br>Zen Pro was used to obtain confocal microscopy (ZEISS) images.<br>OpenEx (OpenWorkbench Version 2.24.0 by Tucker Davis Technologies) was used for Microelectrode array (MEA) reordings.
Data analysis	Cell counting was performed in FIJI ImageJ. Built-in Watershed and cell counter were used to count cells.<br>Optical recordings of Ca2+ indicator were first processed in FIJI ImageJ and then analyzed in Matlab 2020b. (See methods).<br>Alignment analysis was performed in Matlab 2020b (Described in detail in methods section)<br>The cellular aggregation model was developed and analyzed in Matlab 2020b.<br>All plots were created and statistical analysis were performed using Matlab 2020b.<br>All relevant codes are available through corresponding author.<br>An interactive app (.exe and .mlapp versions) with detailed instructions is publicaly available on github at following adddress: https://github.com/mdfayadhasan/Cellular-Aggregation-Model

For manuscripts utilizing custom algorithms or software that are central to the research but not yet described in published literature, software must be made available to editors and reviewers. We strongly encourage code deposition in a community repository (e.g. GitHub). See the Nature Research guidelines for submitting code & software for further information.

Data

Policy information about availability of data

All manuscripts must include a data availability statement. This statement should provide the following information, where applicable:

- Accession codes, unique identifiers, or web links for publicly available datasets
- A list of figures that have associated raw data
- A description of any restrictions on data availability

The data that support the findings of this study are available from the corresponding author upon reasonable request.

Field-specific reporting

Please select the one below that is the best fit for your research. If you are not sure, read the appropriate sections before making your selection.

- [x] Life sciences
- [ ] Behavioural & social sciences
- [ ] Ecological, evolutionary & environmental sciences

For a reference copy of the document with all sections, see nature.com/documents/nr-reporting-summary-flat.pdf

Life sciences study design

All studies must disclose on these points even when the disclosure is negative.

Sample size	3 cultures were analyzed in all experiments
Data exclusions	No data were excluded from the analysis
Replication	Replication in all experiments was confirmed using at least 2 technical and 3 biological replicates.
Randomization	Post-natal day 0-1 rats were chosen randomly irrespective of their gender. Cultures were randomly selected for immunohistochemistry or optical recording from the same experiment. Optical and electrical recordings of all cultures were performed in random order on a given day.
Blinding	Blinding was not possible as the same author acquired and analyzed all data. The analysis however included all acquired data and exact same data analysis algorithms were used to analyze all data groups in each experiment.

Reporting for specific materials, systems and methods

We require information from authors about some types of materials, experimental systems and methods used in many studies. Here, indicate whether each material, system or method listed is relevant to your study. If you are not sure if a list item applies to your research, read the appropriate section before selecting a response.

Materials & experimental systems

n/a	Involved in the study	
☐	☒	Antibodies
☒	☐	Eukaryotic cell lines
☒	☐	Palaeontology and archaeology
☐	☒	Animals and other organisms
☒	☐	Human research participants
☒	☐	Clinical data
☒	☐	Dual use research of concern

Methods

n/a	Involved in the study	
☒	☐	ChIP-seq
☒	☐	Flow cytometry
☒	☐	MRI-based neuroimaging

Antibodies

Antibodies used	The following antibodies were used for immunohistochemistry:<br>Anti-GFAP (1:1000, Abcam, ab7260)<br>Anti-NeuN (1:1000, MilliporeSigma, MAB377)<br>Anti-Beta-3-Tubulin (1:1000, Thermo Fisher Scientific, MA1-118)<br>Goat anti-Rabbit labeled with Alexa Fluor 647 (1:500, Thermo Fisher Scientific, a27040)<br>Goat anti-Mouse labeled with Alexa Fluor 488 (1:500, Thermo Fisher Scientific, a28175)
Validation	All primary and secondary antibodies used in this work are commercially available.<br>Antibodies were validated by the respective manufacturers and this information was provided on their website and product information data sheets

Animals and other organisms

Policy information about studies involving animals; ARRIVE guidelines recommended for reporting animal research

Laboratory animals	Sprague-Dawley rats (Charles River Laboratories), Male and Female, Post-natal day 0-1
Wild animals	This study did not involve wild animals
Field-collected samples	This study did not involve samples collected from the field
Ethics oversight	All animal use protocols were approved by the Institution Animal Care and Use Committee (IACUC) at Lehigh University and were conducted in accordance with the United States Public Health Service Policy on Humane Care and Use of Laboratory Animals.

Note that full information on the approval of the study protocol must also be provided in the manuscript.
